# In Vivo CAR-T Therapies—A New Era of Programmable Immunity

**DOI:** 10.3390/ijms27041737

**Published:** 2026-02-11

**Authors:** Stefano Pierini, Rehman Qureshi, Sergei Pustylnikov, Zhanna Bartosh, Tatiana Akimova

**Affiliations:** 1 Independent Researcher, Philadelphia, PA 19342, USA; rehman.a.qureshi@gmail.com (R.Q.); bakss0312@gmail.com (Z.B.); 2 100XBIO, Inc., Stoneham, MA 02180, USA; sergei@100xbio.com; 3 Division of Transplant Immunology, Department of Pathology and Laboratory Medicine, Children’s Hospital of Philadelphia, Perelman School of Medicine, University of Pennsylvania, Philadelphia, PA 19104, USA

**Keywords:** in vivo CAR-T, CAR-T, lipid nanoparticles, LNPs, T cell engineering, non-viral vectors, gene delivery, gene therapy

## Abstract

Ex vivo chimeric antigen receptor (CAR) T cell therapies have achieved remarkable clinical success over the past decade, enabling effective treatment of several hematologic malignancies once considered incurable. However, their broader use remains limited. Barriers include complex and costly manufacturing, long production timelines, and risk of significant side effects and toxicities, challenges that have been further exacerbated by the reduced investment across the biotech sector since 2022. Emerging in vivo CAR-T approaches seek to overcome many of these limitations by generating CAR-T cells directly within the patient, most commonly using lentiviral or lipid nanoparticles (LNPs) delivery vectors. This strategy has the potential to streamline production, allow more tunable and repeatable dosing, and markedly reduce overall costs. However, it also raises new questions regarding genomic safety, the specificity and durability of CAR expression, host immune responses, pharmacokinetics, and regulatory oversight. In this review, we summarize the major and emerging in vivo CAR-T delivery platforms—analyzing their underlying technology, preclinical and clinical performance, and developmental paths—and discuss the scientific, technical, and biological challenges shaping this rapidly emerging field. We further outline future directions and opportunities in the field of programmable T cell immunity.

## 1. Introduction

Chimeric antigen receptor (CAR) T cell therapy is one of the major breakthroughs in modern cancer immunotherapy. Unlike conventional T cells, which depend on antigen presentation by major histocompatibility complex (MHC) molecules, CAR-engineered T cells express a synthetic receptor that recognizes specific surface antigens on target cells, enabling tumor cell killing in an MHC-independent manner. In B cell malignancies, CD19-targeted CAR-T therapies have revolutionized treatment, with objective response rates of approximately 40–54% in various lymphomas and 71–81% in B cell acute lymphoblastic leukemia [1], often resulting in durable remissions and FDA approvals of multiple products. Emerging clinical evidence also supports CAR-T applications beyond oncology: in refractory systemic lupus erythematosus, CD19 CAR-T therapy has induced profound B cell depletion and clinical remission in early studies [2], and case reports suggest complete remission in severe, treatment-resistant ulcerative colitis following CD19 CAR-T administration [3]. Over the past decades, CAR-T technology has progressed through five generations, with each iteration designed to improve efficacy, persistence, proliferation, and safety [4].

Despite its remarkable clinical success in treating hematological malignancies, current ex vivo CAR-T therapy faces several significant limitations. Excessive expansion or prolonged persistence of CAR-T cells can lead to serious, potentially life-threatening toxicities, including cytokine release syndrome (CRS), immune effector cell-associated neurotoxicity syndrome (ICANS), and immune effector cell-associated hemophagocytic lymphohistiocytosis-like syndrome (IEC-HS) [5]. These and other adverse events may be further exacerbated by lymphodepleting chemotherapy, which is essentially required for effective ex vivo CAR-T therapy [6]. CAR-T manufacturing also presents substantial challenges. Production requires complex, multi-step processing in specialized GMP-certified facilities resulting in high cost, limited availability, and logistical challenges. The interval between leukapheresis and infusion typically ranges from 14 to 21 days or longer. Such delays may be critical for patients with rapidly progressing disease [7]. Another limitation is resistance and relapse. Only a subset of patients achieves long-term remission, even when initial responses are robust. Mechanisms of resistance include CAR-T-intrinsic factors (e.g., exhaustion, poor persistence), tumor-related factors (e.g., antigen escape, immunosuppressive tumor microenvironment), and patient-specific factors (e.g., prior therapies, high tumor burden) [8]. While these resistance and relapse mechanisms have been most extensively characterized in conventional ex vivo CAR-T approaches, they reflect fundamental limitations of CAR-T biology and are, therefore, likely to also impact emerging in vivo CAR-T strategies.

A promising new strategy to overcome many of these limitations is the in vivo generation of CAR-T cells through direct delivery of genetic material to T cells inside the body, using viral or non-viral delivery systems. This approach bypasses the need for lymphodepletion, eliminates specialized cell-manufacturing requirements, and has the potential to make CAR-T therapy faster, more affordable, more accessible, and potentially safer [9]. The ultimate goal of in vivo CAR-T technology is to transform highly individualized cell therapies into broadly applicable, “off-the-shelf” therapeutic products [10,11]. In this review, we summarize recent advances in in vivo CAR-T technologies, compare major and emerging industrial pipelines, and discuss key scientific, technical, and biological challenges, including those unique to the in vivo approach and those inherited from its ex vivo predecessors. To capture the full scope of this rapidly evolving field, we systematically evaluated peer-reviewed literature, conference abstracts, company websites, and press releases, enabling a comprehensive assessment of in vivo CAR-T landscape.

## 2. Delivery Platforms Overview

**Figure 1:**
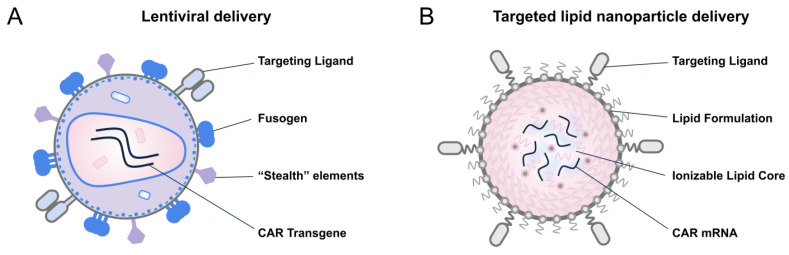
Advanced delivery systems for in situ CAR-T cell engineering. (**A**) Lentiviral-mediated in vivo delivery. The most used viral vectors for in vivo CAR-T generation are engineered lentiviruses optimized for systemic administration and efficient in vivo CAR-T cell generation. Key elements include: Targeting ligand—engineered targeting moieties (e.g., scFv, VHH) that direct the vector specifically to T cell surface receptors (e.g., CD3, CD8 or CD7) and may simultaneously activate T cells (e.g., via CD3 engagement). Fusogen—the viral fusion machinery (e.g., modified VSV-G) that mediates entry and delivery of the viral genome into T cells and controls vector tropism. “Stealth” elements—engineering strategies to reduce clearance (e.g., CD47 overexpression) and immunogenicity (e.g., MHC-I disruption). CAR Transgene (ssRNA)—the diploid genetic blueprint that will be reverse transcribed to integrate as double-stranded DNA into the host genome, and reprogram T cells for durable CAR expression. (**B**) Targeted lipid nanoparticle (LNP)-mediated in vivo delivery. Non-viral platforms frequently utilize targeted LNPs to deliver mRNA to T cells. Key elements are: Targeting ligand—in actively targeted LNPs, ligands (e.g., VHH, scFv, aptamer, or glycans) displayed on the lipid shell to enable binding to T cell surface receptors (e.g., CD3). Lipid formulation—modified lipid components that minimize liver uptake, reduce immunogenicity, and promote endosomal escape. Ionizable lipid core—pH-sensitive matrix that triggers cargo release. CAR mRNA—the functionalized mRNA encoding the CAR construct, often incorporating regulatory elements to enable tissue- or cell-type-restricted activity.

Delivery platforms refer to the carrier systems that transport genetic payload consisting of RNA and/or DNA, into the patient’s T cells in vivo. These platforms can currently be divided into two main categories: viral delivery platforms (e.g., Lentivirus, adeno-associated virus [AAV]), and non-viral delivery platforms (e.g., lipid nanoparticles [LNPs], peptide-based nanoparticles, polymer-based nanoparticles) (Figure 1).

### 2.1. Viral Delivery Platform

**Figure 2:**
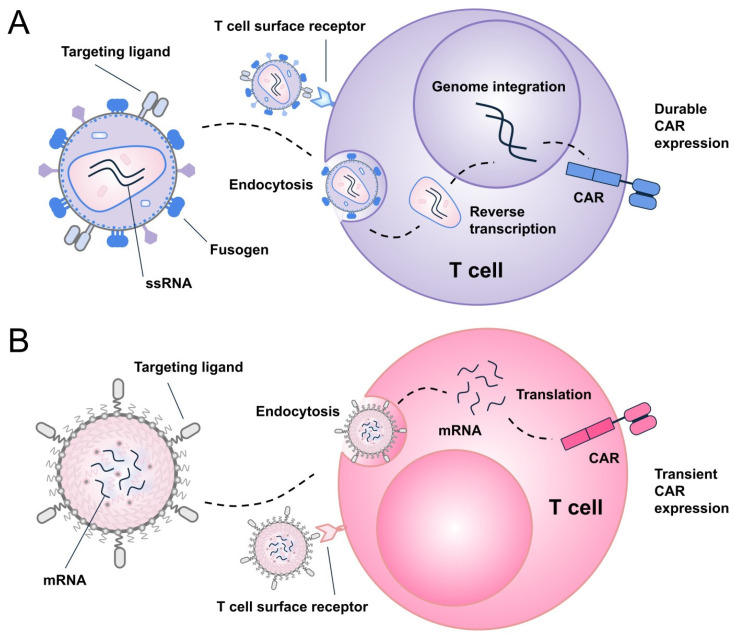
Intracellular mechanisms and expression kinetics of in situ CAR-T engineering. (**A**) Lentiviral transduction pathway. Engineered lentiviral vectors utilize targeting ligands to engage specific T cell surface receptors, triggering endocytosis or direct membrane fusion. Following capsid uncoating, the ssRNA genome undergoes reverse transcription to form double-stranded DNA, which translocates to the nucleus for genome integration. This results in the stable production of CAR proteins, leading to durable CAR expression. (**B**) LNP/mRNA-mediated transfection pathway. Targeted lipid nanoparticles (LNPs) bind to T cell surface receptors to facilitate cellular uptake via endocytosis. Upon endosomal escape, the CAR mRNA is released into the cytoplasm where it is directly accessible to the cellular machinery for translation. Unlike viral delivery, the mRNA does not enter the nucleus or integrate into the host genome. This “hit-and-run” mechanism leads to transient CAR expression, providing a controlled therapeutic window and minimizing long-term safety risks.

Lentiviruses contain a single-stranded RNA genome that undergoes reverse transcription and integration into the host DNA, allowing stable expression of the introduced transgene (Figure 2A).

These vectors are well established for generating ex vivo autologous CAR-T cells, but in vivo use raises added safety concerns. The random genomic integration profile, intrinsic to lentiviral vectors, confers a risk of insertional mutagenesis, making strict control of target cell specificity essential. At the same time, achieving efficient T cell transduction requires avoiding unproductive uptake of vector particles by non-target cells, particularly macrophages, which commonly act as sinks. To address this, lentiviral vectors have been pseudotyped with a variety of viral envelope glycoproteins, including those derived from vesicular stomatitis virus (VSV) [12,13], Nipah virus [14,15,16,17], measles virus [18], cocal virus [19,20], and paramyxoviruses [21,22], among others. These envelope proteins can be further engineered to abrogate interactions with their native receptors, while the incorporation of high-affinity T cell-specific binding domains enables selective T cell targeting [14,15,16,17,18,19,20,23]. VSV glycoprotein (VSV-G) pseudotyped lentiviruses are particularly valued for their high particle stability, enabling efficient vector concentration and high yields. Their pH-dependent fusion within endosomal compartments facilitates highly efficient cellular entry, making VSV-G lentivirus the “workhorse” of gene delivery systems. Because lentiviral particles contain multiple immunogenic proteins that can elicit immune responses detrimental to both the effectiveness and safety of the therapy, strategies aimed at enhancing vector stability in patient serum [24] and minimizing reactogenicity [25] have been adopted by several platforms [26,27].

The first example of lentiviral-based in vivo CAR-T therapy employed a CD8-targeted Nipah virus-pseudotyped vector, which, following systemic administration in humanized mice, induced anti-CD19 CAR-T expression capable of mediating B cell depletion [16]. Restricting CAR delivery to CD4^+^ T cells achieved high efficiency, matching or even surpassing the activity observed with CD8-targeted lentiviral vectors [18]. To enable both T cell engagement and activation, several approaches employ agonistic binders targeting CD3 or the T cell receptor (TCR) [14,23,26], a strategy adopted by most lentiviral platforms discussed in this review, including those developed by Umoja Biopharma (Seattle, WA, USA), EsoBiotech (Mont-Saint-Guibert, Belgium), Kelonia Therapeutics (Boston MA, USA), Shenzhen Genocury (Shenzhen, China), Exuma Biotech (West Palm Beach, FL, USA), Alaya.Bio (Paris, France), and Vyriad (Rochester, MN, USA) (Table 1). In addition to CD3 targeting, Umoja and Genocury have incorporated costimulatory modules into the viral envelope to further potentiate in vivo CAR-T activation, transduction, and anti-tumor efficacy. Conversely, Interius Biotherapeutics (Philadelphia, PA, USA) and Sana Biotechnology (Seattle, WA, USA) utilize anti-CD7 and anti-CD8 binders, respectively, to efficiently target T cells without activation, a feature potentially improving the safety profile (Table 1).

**Figure 3:**
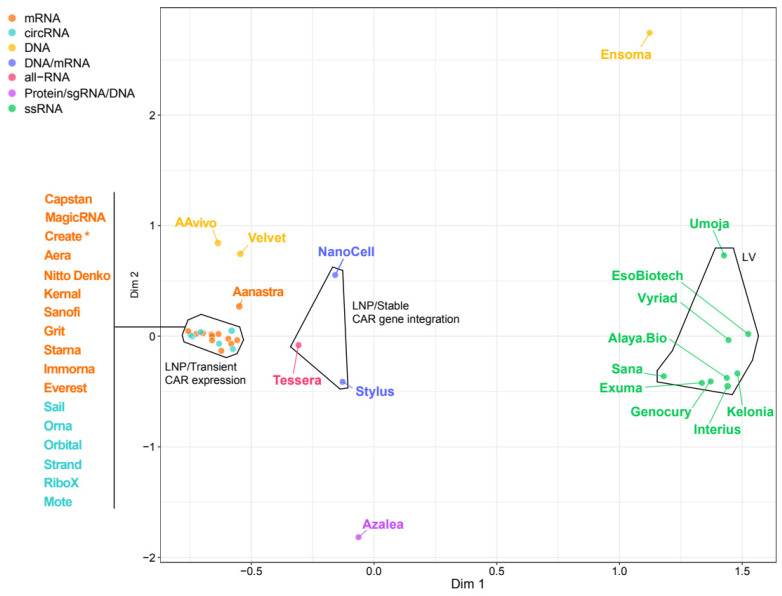
Multiple Correspondence Analysis of delivery platforms. Multiple Correspondence Analysis (MCA) was used as an exploratory approach to visualize similarity and differences across 34 delivery platforms. Each point represents one platform, with greater distances indicating larger differences in technological features, whereas proximity reflects shared characteristics. Points are color-coded by nucleic acid format (see legend). To aid interpretation, three major clusters were manually delineated. These clusters encompass platforms sharing overlapping technological attributes and correspond broadly to LNP-based platforms enabling transient CAR expression (*n* = 17), LNP-based platforms enabling CAR gene integration, supporting durable expression (*n* = 3) and LV-based platforms (*n* = 9). The boundaries of these clusters are illustrative, rather than numerically inferred. Platforms positioned outside these clusters are more differentiated, reflecting unique combinations of features relative to the other platforms. The MCA incorporated a broad set of categorical variables, including delivery system, genome-integration capability, site-specific editing, nucleic acid format, molecular strategy, targeting moiety, inclusion of co-stimulation (LV only), immunogenicity or phagocytosis mitigation strategies (LV only), selection/enrichment mechanisms (LV only), cell-specific promoters (LV only), and source of the native fusogenic glycoprotein (LV only). Dataset is shared in Table A1. Platforms by Cytiva (Marlborough, MA, USA), ImmunoVec (Malibu, CA, USA), Immunofoco (Shanghai, China), Legend (Somerset, NJ, USA), Strm.bio (Cambridge, MA, USA), Carisma (Philadelphia, PA, USA) and Liberate (Boston, MA, USA) are not included. Analyses were conducted in R using the FactoMineR package [29], with slight jitter applied to improve point visibility. * In addition to Create’s (Cambridge, MA, USA) lead programs based on LNP/mRNA, the company is also developing CREATE, which enables site-specific CAR gene integration into T cells (not included).

To overcome limitations inherent to lentiviral platforms, particularly random genomic integration, Azalea Therapeutics (Berkeley, CA, USA) is advancing a dual-vector strategy designed to enable locus-specific CAR insertion in T cells. This hybrid system combines enveloped delivery vehicles (EDV) with adeno-associated virus (AAV) [28], positioning the platform as a clear standout relative to others in the field (Figure 3).

Similarly differentiated, Ensoma Therapeutics (Boston, MA, USA) is pursuing a distinct in vivo engineering strategy that leverages helper-dependent adenovirus platform of virus-like particles (VLP) capable of delivering large genetic payloads into hematopoietic stem cells (HSCs) via CD47 targeting. By pairing this delivery modality with lineage-specific promoters, Ensoma’s platform enables generation of multicellular CAR therapies, an approach that stands apart from other viral delivery systems according to the MCA plot (Figure 3).

**Figure 4:**
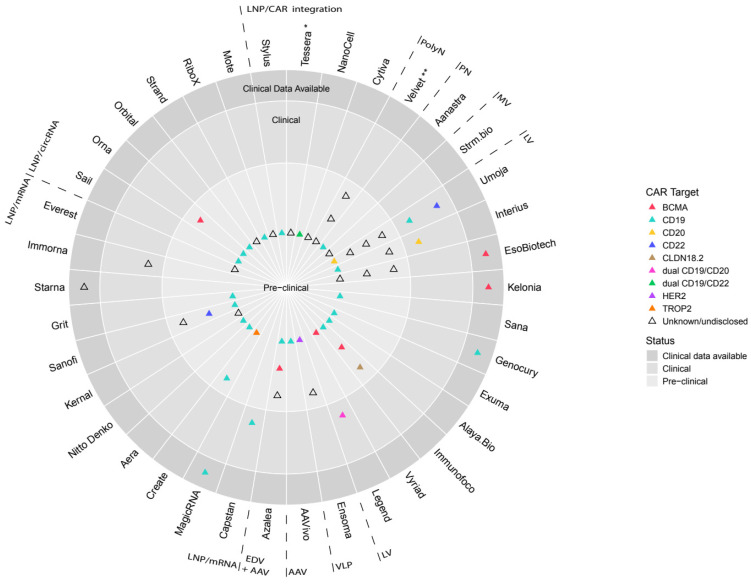
Landscape of in vivo CAR-T programs. The radial plot maps 59 individual programs, each represented by a triangle. Colors denote CAR target antigens (see legend), with 22 programs targeting CD19 (37.3%), 6 BCMA (10.2%), 2 CD20 (3.4%), 2 CD22 (3.4%), 1 dual CD19/CD22 (1.7%), 1 dual CD19/CD20 (1.7%), 1 TROP2 (1.7%), 1 HER2 (1.7%), 1 CLDN18.1 (1.7%), and 22 programs with unknown/undisclosed targets (37.3%). Radial positioning reflects clinical maturity, with 48 assets in preclinical phase, 6 assets in clinical phase, and 5 assets with clinical data available. Outer labels indicate the corresponding companies (*n* = 38) and the delivery modalities, including lentivirus (LV), virus-like particles (VLP), adeno-associated virus (AAV), enveloped delivery vehicle (EDV), LNP/mRNA, LNP circular RNA (circRNA), LNP platforms enabling CAR integration with durable expression (LNP/CAR integration), polymer-based nanoparticle (PolyN), peptide-based nanoparticle (PN), and megakaryocyte-derived extracellular vesicle (MV). * Tessera’s (Cambridge, MA, USA) lead target undisclosed. Anti-CD19, anti-CD20, and anti-BCMA were used as POC (proof of concept) only. ** Velvet’s (Houston, TX, USA) lead targets against solid tumors undisclosed. Anti-CD19 CAR was used as POC only. ImmunoVec, Carisma, and Liberate platforms are not included in the plot.

While initial clinical observations have been described in limited case reports from EsoBiotech, Kelonia and Genocury (Table 1 and Figure 4), comprehensive follow-up and larger patient cohorts will be necessary to establish the most appropriate viral platform for each therapeutic indication.

### 2.2. Non-Viral Delivery Platforms

LNP-based mRNA delivery has emerged as a validated genetic medicine platform, exemplified by the success of COVID-19 vaccines and subsequent FDA approval. Once administered, LNPs are internalized by cells via endocytosis, particularly hepatocytes following intravenous injection. The encapsulated mRNA escapes the endosome, enters the cytoplasm, and is transiently translated before degradation (Figure 2B). Advances in lipid chemistry have led to biodegradable, low-reactogenicity LNP formulations that improve tolerability and enable repeat dosing. Using high-throughput screening, liver-detargeted, immunotropic LNP formulations have been identified, allowing tissue-specific delivery via passive targeting, followed by broad immune cells engineering. For targeted delivery, LNPs can be functionalized with a variety of moieties, including antibodies, antibody mimetics (e.g., designed ankyrin repeat proteins [DARPins]), peptides, sugars (e.g., glycans) or aptamers, directed against T cell markers such as CD8, CD4, CD5, CD7, CD3, or CD2 [30,31,32,33], enabling receptor-specific uptake [34]. Antibodies and antibody fragments (e.g., single-chain variable fragments [scFvs], nanobodies), directed against CD8, represent the most established approach for LNP functionalization (Table 2). While CD3 targeting can induce non-specific T cell activation [31,35] and potentially reduce tolerability, it remains the preferred choice for some LNP platforms discussed in this review (e.g., NanoCell Therapeutics (Utrecht, Netherlands) and Stylus Medicine (Cambridge, MA, USA)) [36], possibly due to its superior targeting efficiency compared to other T cell markers [37]. Nonetheless, ligand conjugation poses challenges related to manufacturing complexity, particle yield, stability, and long-term storage, all of which are being systematically addressed by ongoing industry efforts. Although LNPs have gained immense traction since COVID-19 vaccines, the first evidence supporting non-viral in vivo CAR-T generation came from polymer-based nanoparticle systems [38,39]. These polymeric nanoparticles, typically formulated from cationic biodegradable poly (β-amino ester) polymers, self-assemble with anionic nucleic acids (DNA or mRNA) via electrostatic interactions and undergo pH-dependent endosomal escape following endocytosis. This approach represents an easy-to-manufacture, flexible, and broadly applicable platform for in vivo immune cells reprogramming being advanced by companies like Velvet Therapeutics and ImmunoVec (Table 2). However, polymer-based platform requires further investigation to establish their scalability, efficacy, and safety profile [40] before they can rival LNP-based systems.

Because RNA-based platforms rely on non-integrating, cytoplasmic molecules that are rapidly degraded, LNP/RNA systems are inherently transient, allowing precise dose control. Multiple strategies have been developed to improve mRNA stability and translational performance, notably through optimization of untranslated regions (UTRs) and codon usage [41]. More recently, attention has turned to circular RNA (circRNA)—a covalently closed RNA molecule that lacks free 5′ and 3′ termini. This structure confers resistance to exonuclease-mediated degradation, resulting in greater stability and prolonged intracellular half-life compared with linear mRNA [42]. Synthetic circRNAs can be engineered with internal ribosome entry sites (IRES) or N6-methyladenosine (m^6^A) motifs [43,44,45] to further enhance translation efficiency and reduce immunogenicity. Originally explored for vaccines [46], LNP/circRNA can enable sustained CAR expression and lower dosing frequency compared to conventional mRNA [44,47,48]. Despite challenges, including complex circularization processes that may limit scalability, LNP/circRNA systems are being actively pursued by several biotech companies, including Sail Biomedicines (Cambridge, MA, USA), Orna Therapeutics (Watertown, MA, USA), Orbital Therapeutics (Cambridge, MA, USA), Strand Therapeutics (Boston, MA, USA), RiboX Therapeutics (Shanghai, China), and Mote Therapeutics (Lexington, MA, USA) (Table 2). LNP platforms encapsulating either linear mRNA or circRNA for transient CAR expression currently dominate the non-viral delivery landscape. Many emerging companies have adopted strategies like those used by more established leaders such as Capstan Therapeutics (San Diego, CA, USA) and Shenzhen MagicRNA Biotechnology (Shenzhen, China), resulting in modest differentiation across this portion of the field as reflected by the tight clustering in the Multiple Correspondence Analysis (Figure 3).

While transient and titratable CAR expression can mitigate risks associated with uncontrolled CAR-T expansion and exhaustion, optimal efficacy often relies on repeated dosing. In scenarios where prolonged CAR persistence is desired, LNP-based strategies enabling permanent CAR gene insertion into T cells provide a viable alternative (Figure 3). These elegant knock-in approaches, pioneered by Tessera Therapeutics and further advanced by companies such as NanoCell Therapeutics and Stylus Medicine, differ in their integration precision. Notably, only Stylus platform achieved efficient site-specific payload insertion into a safe-harbor locus, nearly eliminating the risk of insertional mutagenesis (Table 2). Site-specific CAR integration is also being pursued by other emerging LNP platforms such as those from Create Medicines [49], Cytiva [50], Integra Therapeutics (Barcelona, Spain) [51,52]; however, these remain in early developmental stages and have yet to demonstrate in vivo T cell engineering.

## 3. Viral Delivery Platforms Landscape

### 3.1. Lentiviral Vector-Based Platforms

#### 3.1.1. Umoja Biopharma

Umoja’s UB-VV111, is a surface-engineered, third-generation, self-inactivating lentiviral vector which enables efficient in vivo anti-CD19 CAR-T cell generation in preclinical models after a single dose. Currently in clinical testing for relapsed/refractory (R/R) CD19^+^ B cell malignancies (ASH 2024, abstract 1750.1) [53], UB-VV111 is the result of the extensive engineering effort of Umoja’s sophisticated VivoVec platform.

The first VivoVec platform, UB-VV1001 [19], was engineered to display an anti-CD3 scFv for T cell redirection and activation, along with the cocal virus fusion glycoprotein (cocal), which shares similarities with VSV-G, but offers enhanced resistance to human serum inactivation. A second-generation anti-CD19 CAR and a rapamycin-activated cytokine receptor (RACR) are expressed under the control of the MND promoter, a constitutively active hematopoietic lineage promoter [54]. The RACR system is used to selectively expand CAR-T cells: in the presence of rapamycin, non-transduced immune cells are suppressed, whereas in transduced cells, RACR converts rapamycin binding into an IL-2/IL-15-like signaling cascade, providing pro-survival and proliferative cues. In vitro, non-activated PBMCs exposed to UB-VV100 showed dose-dependent T cell activation, transduction, and antigen-dependent cytotoxicity against malignant B cell targets. The resulting CAR T cells exhibited selective expansion in the presence of rapamycin. In vivo, intraperitoneal (IP) administration—to mimic intranodal (IN) injection [55] of UB-VV100 at 0.4 × 10^6^, 2 × 10^6^, and 10 × 10^6^ transducing units (TU) in CD34-humanized mice resulted in a dose-dependent T cell transduction and generated CAR T cells that depleted B cells, reaching B cell aplasia at the highest dose. To evaluate anti-tumor efficacy, Nalm-6 leukemia cells were intravenously injected into PBMC-humanized mice, followed by IP treatment with 27 × 10^6^, 80 × 10^6^ and 270 × 10^6^ of UB-VV100. Across all doses, UB-VV100 was well tolerated, showing no signs of toxicity and induced significant survival benefit [19].

Building on this, a more advanced VivoVec platform [20] was engineered to display a single membrane-bound multidomain fusion (MDF) protein containing the anti-CD3 scFv fused with the active domains of CD80 and CD58 for co-stimulation. This strategy resulted in robust T cell binding and activation. In vivo, PBMC-humanized mice implanted with Nalm6 and treated with MDF VivoVec (10–50 × 10^6^ TU) encoding an anti-CD19 CAR achieved superior T cell activation, accompanied by robust tumor suppression and improved mice survival. The platform was also evaluated in non-human primates (NHP), where a VivoVec vector engineered with a NHP-specific MDF surrogate and encoding an NHP/human cross-reactive anti-CD20 CAR was delivered directly in four animals, three dosed at 2.2–3.8 × 10^9^ TU/animal (2.5 × 10^8^ TU/kg) and one at 0.63 × 10^9^ TU/animal (fivefold lower dose; 0.77 × 10^8^ TU/kg) [20,56]. Remarkably, up to 65% of circulating T cells were CAR-positive by day 10, and B cells were fully depleted by day 7 across all three high-dose animals. In one of those animals, complete B cell aplasia remained until day 76. Transient elevations in C-reactive protein, IL-6, ferritin, and body temperature coincided with CAR-T expansion and resolved as levels declined, indicating a CAR-T-mediated rather than particle-driven response. Liver function remained largely normal, with only minor and short-lived enzyme elevations in a single animal. Biodistribution analysis detected transgenes integration in the injected inguinal and the downstream medial iliac lymph nodes, as well as some low-level transgene signal in the spleen, bone marrow, and lungs. Importantly, transgene integration in the liver, which expresses high level of the cocal ligand low-density lipoprotein receptor (LDL-R) [57], was not observed.

UB-VV111 integrates features of both platforms mentioned above: an envelope displaying the cocal glycoprotein along with the MDF protein, encoding an anti-CD19 CAR with the RACR system. In a GLP toxicity study using CD34-humanized mice, UB-VV111 administered with rapamycin was well tolerated and resulted in CAR T generation and dose-dependent B cell depletion when given IP (surrogate for IN) or intravenously (IV) at up to 5.1 × 10^7^ TU, corresponding to 14 times the highest proposed clinical dose. UB-VV111 vector genome integration was highest in the liver and spleen. Multiplex RNA in situ hybridization method identified murine macrophages and human T cells as the primary transfected cells, while human macrophages transfection was extremely rare, suggesting that murine macrophage transduction is likely a model-specific artifact. Nonclinical toxicology and biodistribution studies in canines demonstrated good tolerability and a favorable biodistribution profile without detectable transduction in any non-lymphoid tissues following IN or IV administrations. In conclusion, given the high potency and low risk of CAR T cell-related toxicity across multiple preclinical models (ASH 2024, abstract 2046) [58], UB-VV111 advanced into the clinic in a first-in-human, dose-finding, phase 1 study (NCT06528301), and it is administered by either the IN or IV route +/− rapamycin in patients with R/R CD19^+^ B cell malignancies [53]. To the best of our knowledge, clinical dose levels have not been publicly disclosed yet. Beyond UB-VV111, Umoja is advancing additional LV-based in vivo CAR-T programs such as UB-VV400/410 (CD22) currently in the clinic (NCT06743503), UB-VV300/310 (CD20), UB-VV500 (target undisclosed), and additional undisclosed products [59,60,61].

#### 3.1.2. Interius Biotherapeutics

Interius’s lead asset INT2104 is non-replicating, self-inactivating lentiviral vector encoding a fully human anti-CD20 CAR that selectively targets human T and Natural Killers (NK) cells and is currently being evaluated in a phase 1 study for relapsed/refractory (R/R) B cell malignancies.

The Interius LV vector platform incorporates a VSV-G-derived Fusogen engineered with a single amino acid substitution (I182E) that abolish native LDL-R binding while preserving fusogenic activity. Two additional substitutions (T214N and T352A), previously shown to confer resistance to human serum and thermal inactivation [24], were also included to yield the optimized Gen 2.1 Fusogen. For cell-specific retargeting, an anti-CD7 binder—engineered with multiple mutations [62,63,64,65] to ablate Fc-mediated effector function and non-specific transduction, was incorporated in the envelope, enabling CD7^+^ T and NK cells transduction while eliminating B cell targeting. Leveraging these advances, INT2104 was generated by combining the Gen 2.1 Fusogen with the CD7-specific binder and an anti-CD20 CAR. Functional studies showed that INT2104-transduced activated PBMCs efficiently lysed CD20^+^ Raji and Daudi B cell lymphoma cell lines and secreted IFN-γ, TNF-α, and IL-2 in an antigen-dependent manner. In CD34-humanized mice, a single intravenous dose of INT2104 (8 × 10^6^ or 2.5 × 10^7^ TU/mouse) led to a marked reduction in circulating B cells by day 7 and complete elimination by day 21. CAR^+^ cells appeared in most treated animals by day 21, coinciding with B cell depletion, and was observed in CD4^+^, CD8^+^ T cells and NK cells. To evaluate in vivo efficacy, immunodeficient mice were implanted with CD20^+^ Raji cells, engrafted with activated PBMCs, and treated with INT2104 at either 1 × 10^7^ TU/mouse (high dose) or 6 × 10^5^ TU/mouse (low dose). High-dose INT2104 fully cleared tumors by day 22, while low-dose treatment achieved complete elimination by day 26. Circulating CAR^+^ cells appeared by day 14 in high-dose groups, while less than half of the mice showed peripheral CAR^+^ cells in the low-dose group. The therapy was well tolerated, with no vector-related weight loss and 100% survival.

As a critical translational bridge between murine models and clinical testing, cynomolgus macaques were employed. Two adult animals received a single intravenous infusion of 2 × 10^9^ TU (~3.5 × 10^8^ TU/kg) of a surrogate vector, identical to INT2104 except for the gag capsid sequence, and were sacrificed on day 4. At necropsy, both treated animals showed marked reductions in circulating B cells. Biodistribution analysis by droplet digital PCR revealed the highest proviral DNA levels in tissues enriched for CD7^+^ cells, including spleen, liver, PBMCs, bone marrow, and lung. Immunofluorescence showed CAR protein expression limited to T and NK cells in the spleen and to T cells in the liver. Importantly, no protein expression was detected in hepatocytes, B cells, or macrophages, although lentiviral particle uptake by liver macrophages was evident by RNAscope method [27]. Additional long-term studies in NHP demonstrated that most macaques experienced B cell depletion within 1–2 weeks post treatment, with gradual recovery to baseline levels by 30–60 days. B cell reconstitution correlated with the development of immune responses against the fully human CAR, suggesting its immunogenicity in NHPs. Notably, one immunodeficient animal exhibited sustained B cell aplasia for one year, with no detectable B cells in blood, spleen, bone marrow, or lymph nodes at necropsy. No CRS, neurological abnormalities, or clinical and hematological changes were observed in toxicology studies (including a GLP study), with follow-up extending to one year for some animals (ASGCT 2024, abstract 2 and 235; Cellicon Valley ’25) [66,67,68].

Based on encouraging preclinical data, Interius has initiated a first-in-human phase 1 trial to evaluate INT2104 (NCT06539338) in B cell malignancies. Beyond its lead program, Interius is developing INT2106, which incorporates an anti-CD19 CAR for severe autoimmune diseases. Another program, INT2108, remains undisclosed with respect to target and indication [69]. Recently, Kite Pharma (Santa Monica, CA, USA), a Gilead Company (Foster City, CA, USA), acquired Interius [70].

#### 3.1.3. EsoBiotech

ESO-T01 is the most clinically advanced example among LV-based platforms, and it is built on EsoBiotech’s proprietary ENaBL platform, a third-generation, non-replicative, self-inactivating, and immune-shielded lentiviral vector system. ESO-T01 encodes a second-generation BCMA-targeted CAR driven by a T cell-specific synthetic promoter which demonstrated promising early clinical data in relapsed/refractory (R/R) multiple myeloma (MM) [26].

ESO-T01 viral envelope incorporates plasma membrane proteins derived from a producer cell line—a MHC-I KO/CD47^hi^/TCR Variable domain of Heavy chain (VHH)-expressing 293T. Key design features include VSV-G mutation to reduce natural tropism, MHC-I deletion to lower immunogenicity [25], CD47 overexpression to evade phagocytosis [71], and a membrane-anchored TCR-targeting VHH nanobody to direct the vector specifically to T cells (CN109134665B) [72]. In preclinical studies [26], using CD34-humanized mice IV engrafted with NCI-H929 MM cells, a single IV administration of ESO-T01 at escalating doses (7 × 10^5^, 1.75 × 10^6^, and 3.5 × 10^6^ TU/mouse) induced dose-dependent expansion of BCMA CAR-T cells, peaking between days 11–20 and reaching ~75% of total CD3^+^ T cells at the highest dose. CAR-T levels gradually declined but remained detectable through day 49. Expansion correlated with tumor clearance, and all treatment groups showed significant tumor suppression and survival benefit. Flow cytometry confirmed transduction specificity, with CAR expression restricted to CD3^+^ T cells and absent in B, NK, or myeloid cells. Biodistribution analysis across 11 tissues detected transgene integration only in the bone marrow, with vector copy number <1.5 copies/cell. The therapy was well tolerated, showing only transient weight loss and mild, self-limited cytokine elevations at the highest dose.

Based on these preclinical results, and notably without preceding NHP studies, a first-in-human, dose-escalation phase 1 study (NCT06691685) was initiated in patients with R/R MM [26]. In July 2025, preliminary data was reported from four patients who received a single infusion of ESO-T01 at a starting dose of 2.0 × 10^8^ TU, corresponding to one-tenth of the human equivalent dose extrapolated from the effective mouse dose (7 × 10^5^ TU/mouse) based on body weight scaling. Immediately after the infusion, all patients experienced CRS, likely from virus-mediated acute immune activation, with grade 3 CRS observed in three patients and grade 1 CRS in one patient. Between days 8 and 12, grade 1 CRS occurred in all patients, with one patient also developing grade 1 ICANS. In this phase, the development of CRS is in line with CAR-T expansion, and symptoms were successfully managed with pharmacological intervention. CAR-T cells were first detected in blood on day 4–8, peaked at day 10–17, and were also found in tumor tissues, pleural effusion, and cerebrospinal fluid. Two patients achieved stringent complete responses by day 28 and 60, while the other two patients showed partial responses with reduction in tumor lesion and minimal residual disease negativity reached in the bone marrow. Together, these early clinical results position ESO-T01 as a promising in vivo CAR-T platform. Nevertheless, a larger cohort and longer follow-up are needed to further determine safety, efficacy, and persistence of ESO-T01.

Beyond MM, EsoBiotec’s pipeline includes exploring ESO-T01 for autoimmune diseases, pending risk–benefit evaluation from the ongoing phase 1 trial. In parallel, the company is advancing a portfolio built on its proprietary ENaBL platform, including ESO-TX101 and ESO-TX102, designed to co-engineer T cells and monocytes, and ESO-TX103, which employs an undisclosed cell-targeting strategy [73]. In March 2025, AstraZeneca (Cambridge, UK) acquired EsoBiotech [74].

#### 3.1.4. Kelonia Therapeutics

Kelonia Therapeutics’ lead clinical candidate, KLN-1010, is an envelope-engineered lentiviral vector encoding a fully human, second-generation anti-BCMA CAR, which, based on early clinical results, enabled four patients with relapsed/refractory (R/R) multiple myeloma (MM) (ASH 2025, LBA-1, AACR 2024, abstract 48) [75,76,77] to achieve minimal residual disease negativity one month after single treatment [77]. Built on the company’s in vivo Gene Placement System (iGPS), KLN-1010 incorporates a proprietary engineered VSV-G Fusogen and anti-CD3 scFv [78] that drive T cell targeting and activation, thereby supporting efficient in vivo CAR-T generation after a single intravenous dose.

Preclinical results demonstrated that anti-BCMA CAR-T cells, generated from in vitro transduced PBMCs, secreted IFNγ upon stimulation with BCMA^+^ cell lines. In vivo efficacy was tested in PBMC-humanized mice bearing subcutaneous BCMA^+^ RPMI-8226 MM tumors, where intravenous KLN-1010 administration elicited a robust, dose-dependent anti-tumor effect. Kelonia’s vector also demonstrated efficacy against large or disseminated (IV-implanted) RPMI-8226 tumors, achieving complete tumor eradication in most animals. In addition, the treatment inhibited growth of the aggressive BCMA^low^ Daudi B cell lymphoma model and, when benchmarked against ex vivo-manufactured CAR-T cell therapy, performed comparably or better. Biodistribution was assessed following infusion of GFP-expressing iGPS particles into PBMC-humanized mice. Among the wide panel of tissues analyzed, only spleen and liver demonstrated notable transduction as indicated by elevated vector copy number. In separate studies with KLN-1010, peripheral CAR expression was detected in human T cells, although murine myeloid cells also showed transient transduction at early time points. Over time, only CAR T cells persisted, becoming the predominant CAR^+^ population. Lineage analysis of peripheral CAR T cells revealed a composition largely enriched in effector and memory CD4^+^ and CD8^+^ subsets. Clinical translatability of the iGPS platform was evaluated in a NHP model, in which animals received doses of 1.25 × 10^8^ or 1.25 × 10^9^ infectious units (IU)/kg of an iGPS surrogate vector encoding an anti-CD20 CAR. Rapid and sustained peripheral B cell depletion was observed within days of administration. B cell reconstitution coincided with the emergence of anti-CAR immune responses around days 35 and 80, indicating immunogenicity against the CAR in NHPs. Robust transduction of PBMCs was detected shortly after vector administration, while vector copy numbers remained below the limit of detection across a broad panel of tissues and organs, suggesting minimal off-target activity. A transient and modest increase in cytokines and chemokines was observed (AACR 2024, poster 48) [76].

Building on these results, Kelonia initiated a phase 1 dose-escalation trial (NCT07075185) to assess the safety, tolerability, pharmacology, and preliminary efficacy of a single administration of KLN-1010 in MM patients. Early clinical observations are promising: all four treated patients achieved minimal residual disease-negative responses by one month, with one sustained for up to five months. The first three patients received 2 × 10^7^ IU/kg, while the fourth was treated at a dose of 6 × 10^6^ IU/kg. CAR-T expansion was consistent with the levels seen in approved ex vivo products, with CAR-T becoming detectable from day 15 and a predominance of memory-phenotype T cells. Treatment was well tolerated with CRS successfully managed with dexamethasone and tocilizumab. No neurotoxicity was observed [77]. These findings point to meaningful early efficacy and a manageable safety profile, with ongoing follow-up required to confirm durability and long-term outcomes. Beyond KLN-1010, Kelonia demonstrated the use of iGPS for in vivo B cell engineering (ASGCT 2025, abstract 1281) [79], and the company’s pipeline currently shows two undisclosed programs. In November 2025, Kelonia entered a collaboration with Jonhson & Jonhson (New Brunswick, NJ, USA) to advance the discovery of novel in vivo CAR-T therapies [80].

#### 3.1.5. Sana Biotechnology

The company’s lead in vivo CAR-T candidate is a CD8-targeted lentiviral vector encoding an anti-CD19 CAR, designed to reprogram T cells in vivo with a single dose and, it is currently in preclinical development for oncology and autoimmune indications.

Sana’s LV vector platform is based on a third-generation self-inactivating lentiviral vector equipped with an engineered Fusogen derived from paramyxoviruses [81]. In this system, the Fusogen’s native receptor-binding domain is disabled and replaced with a T cell-specific binding domain. Upon engagement with its receptor, the engineered Fusogen triggers direct fusion with the cell membrane, enabling cytoplasmic delivery of the payload and thereby circumventing potential non-specific uptake associated with endosomal entry in VSV-G-based vectors. Sana’s early work with the CD8-targeted lentiviral vector in Nalm-6 tumor bearing mice demonstrated robust production of CD8 CAR-T cells, leading to tumor eradication (ASGCT 2022, abstract 1081) [82]. In NHP, in vivo delivery of an anti-CD20 CAR using a CD8-targeted LV vector specifically transduced T cells, notably without inducing their activation, while still achieving robust B cell depletion. No vector or CAR-T related toxicity were observed (ASH 2021, abstract 2769, ASGCT 2023, abstract 760) [83,84]. Beyond in vivo CAR-T, Sana’s pipeline includes a stem cell-derived pancreatic islet cell program for type 1 diabetes (SC451) [85].

#### 3.1.6. Shenzhen Genocury

Shenzhen Genocury, in collaboration with the First Affiliated Hospital of Zhengzhou University, is developing JY231—a CD3-targeted lentiviral vector displaying an undisclosed activation domain [78] for in vivo generation of anti-CD19 CAR-T therapy. The company reported data from a single patient with relapsed/refractory (R/R) diffused large B cell lymphoma who achieved complete remission one month after treatment, with a durable response sustained for over three months (SITC 2024, abstract 1482) [86,87]. CAR-T cell expansion peaked in vivo on day 17 post-treatment and the patient experienced only myelotoxicity, grade 1 CRS, and no neurotoxicity. Unlike other in vivo CAR-T approaches, in this case the patient underwent apheresis to collect T cells, followed by lymphodepletion conditioning and co-infusion of autologous T cells with the lentiviral vector [87]. The inclusion of T cell co-infusion and lymphodepletion, unusual for an in vivo CAR-T approach, was likely intended to enhance therapeutic potency in this patient. Although these results are encouraging, it remains unclear how the trial will proceed and whether future patients will receive T cell co-infusion or lymphodepletion. Notably, the protocol listed on ClinicalTrials.gov (NCT06678282) does not include T cell infusion [88], and a recent company claim suggests that JY231 does not require lymphodepletion [89].

#### 3.1.7. Exuma Biotech

This company is pursuing a CD3-targeted lentiviral vector encoding an anti-CD19 CAR for in vivo CAR-T engineering with potential applications in autoimmune conditions. In CD34-humanized mice, IP administration of Exuma’sLV vector at doses of 1 × 10^6^, 1 × 10^7^, or 5 × 10^7^ TU resulted in dose-dependent CAR expression accompanied by reductions in circulating human B cells. The 1 × 10^7^ TU IP dose led to marked depletion of B cells across peripheral blood, intraperitoneal fluid, bone marrow, and spleen, while complete splenic B cell ablation was achieved only at the 5 × 10^7^ TU IP dose. When injected IV at 1 × 10^7^ TU, the therapy produced significant B cell clearance (AACR 2023, abstract 2918) [90], underscoring its clinical relevance. Beyond its LV-based in vivo CAR-T program, the company is developing an LNP/mRNA in vivo CAR-T and an autologous logic-gated CAR-T platforms with tumor microenvironment-restricted activity [91].

#### 3.1.8. Alaya.Bio

Scientists at Alaya.Bio are advancing a VSV-G-deficient lentiviral vector platform, encoding an anti-CD19 CAR and coated with poly β-amino ester biodegradable polymers along with a polysarcosine lipid-shielding polymer allowing the bioconjugation with CD3-targeting agents. The VSV-G-deficient vector is non-infective until coated with pH-responsive cationic polymers, while the shielding polymer enhances stability. In vitro, poly β-amino ester-based vectors achieved durable, dose-dependent transgene expression and integration in non-activated primary T cells. In vivo, repeated intravenous dosing in mice showed a strong blood-cell tropism, suggesting a distinct biodistribution compared to VSV-G-pseudotyped LVs, and long-term expression of transgenes in blood leukocytes for up to 80 days. Repeat dosing was well tolerated with no signs of toxicity, and efficacy studies showed B cell depletion in healthy immunocompetent mice and tumor regression in A20 lymphoma tumor-bearing mice (ASGCT 2025, abstract 1213 and 1214) [92,93].

#### 3.1.9. Shanghai Immunofoco Biotech Research

This company is driving the development of a novel lentiviral vector pseudotyped with an engineered MxV glycoprotein and equipped with a T cell-targeting ligand for in vivo CAR-T generation. MxV glycoprotein exhibited markedly higher efficiency than conventional VSV-G-pseudotyped vectors, achieving superior T cell transduction. Engineered MxV glycoprotein mutants were rationally designed to reduce infectivity in non-T cells, while incorporation of a T cell-specific module restored selective transduction. Employing this platform, the company achieved complete tumor eradication after one IP injection of the vector one day post PBMC infusion in Nalm6-bearing mice, accompanied by robust CAR-T expansion (ASGCT 2025, abstract 792) [94]. Although the CAR target was not disclosed in the above mentioned study, a recent update highlighted three distinct preclinical in vivo CAR-T programs: one targeting CD19 for diffuse large B cell lymphoma (DLBCL), one BCMA for multiple myeloma and one CLDN18.2 for solid tumor [95].

#### 3.1.10. Vyriad

Vyriad recently announced its entry into the in vivo CAR-T cell therapy field through a collaboration with Novartis [96]. Shortly thereafter, the company reported the preclinical development of a CD3-targeted lentiviral vector, LV-169, encoding a second-generation anti-BCMA CAR under the control of a T cell lineage-specific promoter. LV-169 is pseudotyped with a combination of chimeric and non-chimeric VSV-G proteins that are detargeted from the LDL-R and retargeted to T cells via a CD3-specific scFv. Human T cells expressing Vyriad’s anti-BCMA CAR demonstrated efficient killing of BCMA-positive myeloma target cells. In PBMC-humanized mice, a single intravenous administration of LV-169 at varying doses, resulted in dose-dependent clearance of disseminated OPM-2 multiple myeloma within 28 days. Eighty-four days post-treatment, mice remained tumor-free and were resistant to rechallenge with OPM-2 tumor cells. Circulating CAR-T cells, monitored by flow cytometry, became detectable by day 14 and peaked between days 21 and 35 post-injection, followed by a marked contraction after tumor clearance. Treatment was well tolerated, and cytokine profiling confirmed the absence of severe CRS (ASH 2025, abstract 5896) [97].

Beyond LV-169’s underlying technology, Vyriad has developed additional retargeting strategies to selectively transduce resting T cells within lymphoid tissues. Vyriad’s programs include oncolytic virotherapy, in vivo gene therapy, and gene editing programs, with multiple assets currently in phase 1–2 oncology trials [98].

#### 3.1.11. Legend Biotech

Legend is one of the latest players to announce its entry in the in vivo CAR-T race, and it recently revealed that its lead in vivo CAR-T program is a lentiviral-based platform delivering a dual anti-CD19/CD20 CAR to T cells. Using this approach, Legend has initiated treatment of patients with non-Hodgkin lymphoma (NHL) in an early-stage clinical study [99,100]. Further details on the vector or the dose have not yet been disclosed. Beyond its in vivo CAR-T efforts, Legend’s often underappreciated pipeline includes multiple clinical-stage ex vivo autologous CAR-T programs, as well as an allogeneic CAR-T program [101].

### 3.2. Adeno-Associated Virus (AAV)-, Enveloped Delivery Vehicle (EDV)- and Virus-Like Particle (VLP)-Based Platforms

#### 3.2.1. Ensoma

This company is pioneering an elegant approach to generate in vivo multicellular anti-HER2 CAR therapies, including CAR macrophages and monocytes (CAR-M), CAR-T and CAR-Natural Killers (CAR-NK). Currently, in preclinical development for solid tumor applications, Ensoma’s uniquely diversified technology utilizes proprietary helper-dependent adenovirus platform of virus-like particles (VLPs).

Ensoma’s platform centers on in vivo engineering of HSCs using liver-detargeted VLPs that selectively bind human CD46, a receptor highly expressed on primitive HSCs [102,103]. These VLPs lack viral genes, reducing immunogenicity and supporting large payloads of up to 35 kb, sufficient to accommodate complex genetic cassettes. Co-delivery of a hyperactive Sleeping Beauty transposase (SB100X) enabled stable genomic integration of anti-HER2 CAR constructs expressed under lineage-specific promoters, together with an EF1⍺-driven MGMT^(P140K)^ cassette for chemotherapy-based enrichment of gene-modified HSCs. To achieve selective payload expression, Ensoma identified and validated lineage-restricted promoters with myeloid or T/NK cell-specific activity, successfully generating functional CAR-M, CAR-T, and CAR-NK cells. To assess VLPs’ ability to generate CAR^+^ cells in vivo, hCD46 knock-in mice underwent HSC mobilization followed by intravenous VLP administration. After a one-week rest period to allow engineered HSCs to home and engraft in the bone marrow, mice received chemotherapy to enrich for CAR^+^ immune cells. Eight weeks post VLP administration, flow cytometry revealed approximately 30% CAR-M, 15% CAR-NK cells, and 3% CAR-T cells in the periphery, confirming in vivo CAR engineering. To evaluate the anti-tumor activity of multiplexed CAR-M/NK/T cells, bone marrow from mobilized, VLP-treated, and chemotherapy-conditioned donor mice was transplanted into recipient C57Bl/6 mice orthotopically implanted with HER2^+^ EO771 tumor. VLPs encoding myeloid- and T/NK-restricted promoters induced lineage-specific CAR^+^ immune cells capable of tumor infiltration and tumor growth control (ASGCT 2025, Poster 1783; SITC 2025, abstract 302) [104,105].

Collectively, Ensoma’s platform enables scalable and durable in vivo engineering of multiple immune effector cell types, with the potential to deliver long-lasting anti-tumor activity. Beyond the company’s solid tumor effort, Ensoma’s pipeline includes in vivo CAR-T for heme-oncology along with other programs for chronic granulomatous disease and sickle cell disease [106].

#### 3.2.2. AAVivo/Virovek

A collaboration between those two companies, AAVivo (Houston, TX, USA) and Virovek Incorporation (Houston, TX, USA), represents one of the few reported efforts leveraging adeno-associated virus (AAV) vectors for in vivo CAR-T cell generation. Their lead candidate, AVO-100, is a targeted AAV platform designed to deliver an anti-CD19 CAR directly to T cells, currently in preclinical development. To overcome the intrinsically low efficiency of AAV-mediated T cells transduction, a capsid-engineering strategy was developed in which the VP1, VP2, and VP3 proteins are genetically modified to alternative AAV cell tropism, while the incorporation of T cell-targeting moieties enables selective T cell transduction. This approach supports effective transduction of non-activated T cells while maintaining efficient vector production. Preclinical in vitro testing demonstrated that AVO-100 transduced human PBMCs, achieving CAR expression in 42–54% of T cells, and near complete B cells depletion within 72 h. In a coculture assay with CD19^+^ Raji cells, AVO-100-generated CAR-T cells mediated robust and specific tumor cell killing. In vivo, IV administration of Raji cells in mice, followed by human PBMC engraftment and AVO-100 treatment, significantly reduced tumor burden and improved survival. At study end, 0.3–69.9% of peripheral human T cells expressed the CAR, highlights considerable variability (ASGCT 2025, abstract 1739; SITC2025, abstract 1001) [107,108]. Beyond in vivo CAR-T, AAVivo is advancing an AAV-based therapy to selectively eliminate malignant cells and a platform designed to induce in situ production of multi-specific T cell engagers [109].

#### 3.2.3. Azalea Therapeutics

Azalea’s ground-breaking technology leverages a proprietary dual-vector strategy for locus-specific CAR gene insertion into T cells and is advancing an anti-CD19 in vivo CAR-T therapy for B cell malignancies and autoimmune diseases. Azalea’s core technology, the enveloped delivery vehicle (EDV), employs membrane-derived particles coated with antibody fragments to recognize cell-specific antigens and transiently deliver CRISPR–Cas9 ribonucleoproteins. These Cas9 EDVs exploit antibody–antigen interactions to enable selective genome editing in target cells while minimizing off-target effects and bystander editing. When multiplexed to engage T cell markers, Cas9 EDV technology efficiently generated genome-edited CAR-T cells in humanized mice [110]. To achieve site-specific gene insertion, Azalea combines this EDV system with a T cell-tropic adeno-associated virus to deliver a promoterless homology-directed repair template, enabling CAR payload insertion at defined genomic site within the T cells [28]. Although this elegant engineering strategy claims enhanced precision, durability, and safety, the demonstration of efficient site-specific T cell genome editing in vivo has yet to be demonstrated. Beyond its CD19-based program, the company is progressing a BCMA-targeted in vivo CAR-T for multiple myeloma and an undisclosed program for solid tumors [28].

## 4. Non-Viral Delivery Platform Landscape

### 4.1. LNP/mRNA Platforms Enabling Transient CAR Expression

#### 4.1.1. Capstan Therapeutics

Scientists at Capstan Therapeutics and the University of Pennsylvania are advancing CPTX2309, a CD8-targeted LNP/mRNA-delivered in vivo anti-CD19 CAR-T therapy currently in phase 1 for the treatment of B cell-mediated autoimmune diseases. Capstan’s next-generation LNP formulation contains a novel ionizable lipid, Lipid 829 (L829), designed to reduce liver delivery and enhance immune cell targeting. In mice, CD5-targeted L829-LNP encapsulated with reporter mRNA showed spleen-specific targeting and no liver expression. To assess safety and tolerability, LNP-sensitive species like Sprague-Dawley rats and cynomolgus monkeys [111] were treated with CD5-L829-tLNP. Compared to conventional CD5-tLNP, this next-gen formulation induced lower acute phase proteins and liver enzymes, indicating improved safety and tolerability [41].

To generate potent CD8 CAR-T cells, CD8-tL829-LNPs were encapsulated with UTR- and codon usage-optimized anti-CD19 CAR mRNA. In PBMC-humanized mice, a single 30 µg dose of CD8-L829-tLNP-CD19 induced complete B cell depletion within 3 h, with CAR expression peaking at 6 h and remaining detectable for 24 h. In CD34-humanized mice, three doses of 30 µg administered every 3 days resulted in rapid and sustained B cell depletion for up to 14 days. In a Nalm6 tumor model, PBMC-humanized mice treated twice weekly with 10 or 30 µg for five doses showed a robust, dose-dependent anti-tumor response, achieving near-complete tumor clearance in the high-dose group by day 6 [41].

To evaluate this in a more translationally relevant model, surrogate CD8-L829-tLNPs, encoding a NHP cross-reactive anti-CD20 CAR, were administered in cynomolgus monkeys every three days for three times with increasing doses of tLNP, ranging from 0.1 to 2.0 mg/kg. A sharp decline of B cells in blood, which approached undetectable levels by 24 h, was observed across all doses. Recovery began around day 21 and B cells returned to near-baseline by day 35, displaying a predominantly naïve phenotype, consistent with immune reset. CAR expression appeared dose-dependent, with up to 85% of CD8^+^ T cells and up to 95% of CD8^+^ NK cells expressing the CAR after the third dose. Mild transient increases in body temperature along with C-reactive protein levels, liver enzymes, and cytokines were observed. One high-dose (1.5 mg/kg) animal developed an immune effector cell-associated hemophagocytic lymphohistiocytosis-like syndrome after the third dose, a known CAR-T toxicity [112], and was humanely euthanized. To better define the therapeutic window, additional treatments were tested as follows. A shortened two-dose regimen achieved comparable efficacy to that seen with three doses, but with fewer cytokine elevations. Premedication with corticosteroids and antihistamines—commonly administered to patients receiving intravenous LNP products [113,114]—maintained CAR-T generation and B cell depletion while reducing IL-6 and CRP [41].

Based on these promising data, Capstan initiated a first-in-human phase 1 study (NCT06917742) to evaluate CPTX2309 for the treatment of B cell-mediated autoimmune disorders in healthy volunteers [115]. In the summer of 2025, Amgen (Thousand Oaks, CA, USA) acquired Capstain [116].

#### 4.1.2. Shenzhen MagicRNA Biotechnology

MagicRNA is also advancing an engineered CD8-tLNP/mRNA encoding an anti-CD19 CAR (referred to as HN2301), currently being tested in an early-phase clinical trial (NCT06801119) for relapsed and refractory (R/R) systemic lupus erythematosus (SLE). Early clinical results from five patients indicated that HN2301 generated functional anti-CD19 CAR T cells capable of depleting B cells and reducing disease activity [117], providing the deepest clinical experience today in this category.

HN2301 incorporates proprietary antibody fragments, ionizable lipids, and functional RNA. Incubation of HN2301 with PBMCs led to high CAR expression (>70% at 72 h) on CD8 T cells followed by complete B cells depletion in both healthy donors and SLE patients. Using a surrogate tLNP/mRNA anti-CD20 NHP-cross-reactive CAR, cynomolgus macaques were treated with 0.2 or 0.5 mg/kg for 1, 2, or 3 doses every 2 days. Three days after the final treatment, CAR expression was detected primarily on circulating CD8^+^ T cells with peak transfection reaching ~30% at 0.5 mg/kg dose, and to a lesser extent in monocytes. Correspondingly, significant B cell reductions were observed in blood, spleen, bone marrow, and lymph nodes after each dose, with complete depletion achieved following three 0.2 mg/kg doses or two 0.5 mg/kg doses. Peripheral B cells recovered within 10 days of LNP administration, and the repopulated cells were predominantly naïve, consistent with a B cell reset. No obvious toxicities were observed, aside from a transient, dose-dependent elevation in IL-6 (ASGCT 2025, abstract 794) [117,118].

Building on preclinical success, a dose-escalation study in patients with R/R SLE, treated one, two or three times with 2 or 4 mg/kg of HN2301 every 2 days was initiated. CAR-T cells peaked 6 h after infusion, reaching ~60% of CD8^+^ T cells in patients receiving 4 mg/kg, and returned to baseline within 2–3 days. Off-target CAR expressions in non-CD8^+^ T cells remained below 10%. Circulating B cells declined markedly within 6 h of the first treatment—substantially reduced at the 2 mg dose and fully depleted at the 4 mg dose—with depletion sustained for 7–10 days. Therapy was well tolerated with only low-grade CRS and no clinically significant liver enzyme elevations or cytopenias. In two patients receiving multiple 4 mg/kg doses, anti-nucleosome and anti-dsDNA antibody levels decreased substantially, accompanied by normalization of low complement levels. At 3 months post-treatment, all five patients showed reduced disease activity [117]. Although promising, more data and longer follow-up are necessary to determine optimal dose required to achieve long-term, drug-free remission. Beyond HN2301, an additional program for autoimmune conditions, HN2401, is currently listed on the company pipeline [119].

#### 4.1.3. Create Medicines

Create Medicines recently announced in vivo CAR-T programs for B cell-mediated disorders in malignant hematology and autoimmunity, and a multi-lineage CAR therapy targeting solid tumors. Formerly known as Myeloid Therapeutics (Cambridge, MA, USA), the company initially focused exclusively on LNP/mRNA in vivo CAR-M therapies such as MT-303 (anti-GPC3 CAR) (SITC 2024, abstract 1125) [120] and MT-302 (anti-TROP2 CAR) (SITC 2025, abstract 1342) [121,122], which entered clinical evaluation for hepatocellular carcinoma and epithelial malignancies [78], respectively. The company has since extended preclinical proof-of-concept to HER2-directed in vivo multicellular CAR-M and CAR-NK (MT-304) therapy for solid tumors (ASGCT 2025, abstract 1727) [123].

Using similar LNP-based platforms, Create expanded its focus to include in vivo T cell reprogramming with CRT-401, a dual LNP/mRNA therapy consisting in one product co-formulated with a myeloid/NK-directed anti-HER2 CAR and a T cell-targeted anti-TROP2 CAR. Moreover, the company is advancing CRT-402, a CD8 tLNP/mRNA-delivered anti-CD19 CAR-T for autoimmune conditions. Preclinical evaluation of CRT-402 showed robust Nalm-6 tumor regression in mice after repeated administration. In NHP, two doses (0.75 mg/kg) of tLNP/mRNA encoding an anti-CD20 CAR resulted in profound B cell depletion across blood, spleen, bone marrow, and lymph nodes [124]. In addition to CRT-402, Create is developing a platform for the generation of durable in vivo anti-CD19 CAR-T cells for hematological malignancies, enabled by site-specific transgene insertion into T cells. Toward that goal, the company recently introduced CREATE, a CRISPR-enabled Autonomous Transposable Element platform, designed to achieve site-specific gene integration without DNA double-strand breaks [49,125]. This all-RNA gene-editing technology leverages the human LINE-1 (L1) retrotransposon, the only active autonomous “jumping gene” in the human genome, whose encoded proteins (ORF1p and ORF2p) mediate reverse transcription and genomic integration of RNA cargo. In CREATE, modified L1 mRNA carries a therapeutic payload, while a CRISPR-based nickase guides targeted insertion through L1-mediated reverse transcription and integration. Using this system, an anti-CD19 CAR gene was successfully inserted into a safe-harbor locus and expressed in primary human T cells in vitro. These engineered CAR-T cells exhibited activation, cytokine release, and selective cytotoxicity upon coculture with Nalm6 target cells. CREATE represents an elegant tool for precise genome engineering and supports a path for stable, site-specific in vivo T cell editing. At present, however, evidence of successful in vivo modification using this system is lacking.

#### 4.1.4. Aera Therapeutics

Aera Therapeutics (Cambridge, MA, USA) is also developing a tLNP/mRNA-delivered anti-CD19 in vivo CAR-T therapy for autoimmune disease. Its lead candidate, AERA-109, has shown robust B cell depletion in blood and tissue of both mouse and NHP models following a short two or three treatment cycle (ASGCT 2025, poster 770) [126].

Functionalized with a CD8-targeting moiety, Aera’s proprietary LNPs composition incorporated novel ionizable lipids enabling selective CD8^+^ T cell transfection while minimizing liver tropism. A second-generation anti-CD19 CAR is transiently expressed by optimized mRNA, which has been extensively engineered (CAR domains, codon usage, UTRs, 5′ cap, poly(A) tail, etc.) to maximize translation and therapeutic efficacy. Leveraging this technology, Aera demonstrated dose-dependent CAR expression in non-activated human T cells, which eradicated CD19^+^ Nalm6 upon co-culture. Similarly, in vitro transfection of PBMCs resulted in dose-dependent B cell depletion and T cell activation, confirming antigen-dependent functionality. In vivo, a single injection of GFP-encoding tLNP (1 mg/kg) in tumor-free CD34-humanized mice drove selective expression in CD8 T cells, reaching up to 80% in spleen and blood, 60% in bone marrow, and 20% in lymph nodes, with minimal expression in other immune subsets. In a separate experiment using the same model, two doses of LNP/mRNA-encoded anti-CD19 CAR achieved significant dose-dependent CAR expression in CD8^+^ T cells (~45% at 1 mg/kg) along with B cell depletion in blood and spleen. Having demonstrated selectivity and efficacy in mice, Aera’s platform was then tested in NHPs using a surrogate anti-CD20 CAR mRNA (0.25 mg/kg per dose, every 3 days, 3 times). Robust B cell depletion was observed in blood 1 day after the first dose and was sustained throughout the 10-day study period. Blood mRNA levels of the CAR gene showed consistent pharmacokinetics with no evidence of accelerated CAR clearance upon repeat dosing [126]. In a separate study, NHPs received two doses of 0.3 mg/kg administered three days apart. Although anti-CD20 CAR-T level did not surpass 5% in blood, peripheral B cell depletion was evident within 24 h of the first dose, with complete elimination observed in the spleen and bone marrow by Day 8 (10th Annual CAR/TCR Summit; ASH 2025, abstract 4106) [127,128]. Beyond AERA-109, the company is also advancing a diverse preclinical pipeline, including next-generation tLNPs, antibody–oligonucleotide conjugates for cardiac and muscle targets, and protein nanoparticles for central nervous system disorders.

#### 4.1.5. Nitto Denko Corporation

Scientists at Nitto Denko (Osaka, Japan) are advancing an anti-CD8 VHH-targeted LNP delivering anti-CD19 CAR mRNA. Optimization efforts revealed a critical dependence on VHH ligand density for efficient delivery and identified an optimal density that maximized in vitro reporter expression. In vivo, this optimal configuration achieved selective mRNA delivery to CD8^+^ T cells with minimal off-target uptake in CD4^+^ T cells, Tregs, or B cells. When formulated with anti-CD19 CAR mRNA, these LNPs induced dose-dependent CAR expression, B cell depletion, and anti-tumor efficacy in Nalm6-bearing PBMC-engrafted mice at doses ranging from 0.5 to 2.0 mg/kg, as well as splenic B cell depletion at lower doses between 0.05 and 0.25 mg/kg. (ASGCT 2025, abstract 665) [129].

#### 4.1.6. Kernal Biologics

Kernal’s (Cambridge, MA, USA) T cell-targeted LNP/mRNA in vivo CAR-T platform is currently in preclinical testing for B cell-mediated autoimmune diseases and hematologic malignancies. The platform integrates advanced engineered mRNA payloads, optimized for enhanced T cell-selective transduction, with proprietary LNP formulation designed to redirect biodistribution away from the liver and toward secondary lymphoid organs. Using this platform, reporter expression was observed in nearly all CD4^+^ and CD8^+^ T cells (~90%), achieving >20-fold higher expression in T cells compared to myeloid cells and >60-fold compared to B cells. In vivo, intravenous administration of tLNP delivering murine CAR mRNA in C57BL/6 mice led to rapid depletion of peripheral B cells within days, confirming platform efficacy in vivo (ASGCT 2025, abstracts 666 and 1753) [130,131]. Recently Kernal announced its intention to advance KR-402, its lead in vivo CAR-T candidate, toward clinical development for multiple sclerosis and B cell malignancies, including acute lymphoblastic leukemia, large B cell lymphoma, and chronic lymphocytic leukemia [132]. Beyond KR-402, the company’s pipeline includes three additional undisclosed programs for solid tumors [133].

#### 4.1.7. Sanofi

Sanofi (Paris, France) has reported preclinical results for its in vivo CAR-T programs using proprietary CD8-targeted LNP/mRNA encoding anti-CD22 or anti-CD19 CARs for oncology and autoimmune indications.

The optimized LNP formulation, identified through extensive ionizable lipid screening, demonstrated improved tolerability and stability. Efficient T cell transfection required incorporation of a CD8-targeting moiety, as untargeted LNPs failed to transfect T cells [78]. In vitro, CD8^+^ T cells exposed to CD8-tLNP/mRNA encoding anti-CD22 CAR achieved transfection rates of up to 80%, with durable CAR expression and effective Nalm6 cell killing, without non-specific activation. CAR expression in whole-blood assays was restricted to CD8^+^ T cells and NK cells. In PBMC-humanized mice, repeated dosing induced CAR expression in >80% of circulating CD8^+^ T cells, resulting in complete B cell depletion and anti-tumor activity without systemic toxicity in a B cell tumor model (ASH 2024, abstract 3421) [134]. More recently, an anti-CD8 nanobody-targeted LNP encoding an anti-CD19 CAR was evaluated in rhesus macaques. Single or repeated dosing was well tolerated, with rapid CAR expression detectable within 1 h, peaking at 24 h, and associated with up to 95% peripheral B cell depletion. CAR-expressing CD8^+^ T cells and NK cells were detected in bone marrow, accompanied by >90% B cell depletion, with gradual recovery by days 29–50 (ASGCT 2025, abstract 260) [135]. In conclusion, these results provide preclinical evidence that Sanofi’s LNP/mRNA platform can effectively deplete endogenous B cells and control tumor growth. Although plans to initiate clinical development have not yet been disclosed [78], the company announced its intention to advance three in vivo CAR-T programs [136].

#### 4.1.8. Grit Biotechnology

Grit (Shanghai, China) is developing a T cell-targeted LNP/mRNA therapy designed to deliver anti-CD19 CAR in vivo for the treatment of autoimmune diseases. The company platform uses controllable ligand attachment modification and purification technology to streamline the surface conjugation and purification of antibody-functionalized LNPs. This technology enables precise control of linker chemistry and antibody engineering, ensuring efficient and reproducible particle modification. Using this system, Grit’s LNPs, functionalized with the optimal T cell target (undisclosed), enabled over 30% CAR expression in CD3^+^ T cells, with preferential transfection of the CD8^+^ subset and near-complete B cell killing within 24 h in vitro. In vivo, a single dose (0.33 mg/kg) of tLNP delivering anti-CD19 CAR mRNA induced >95% B cell depletion in the spleen of humanized mice. In a Nalm-6 leukemia model, both 0.2 and 1 mg/kg doses achieved complete tumor control, with full eradication of large, established tumors at 1 mg/kg (ASGCT 2025, abstract 1282) [137]. In addition to its preclinical in vivo CAR-T program, Grit is also advancing APC-targeted neoantigen mRNA vaccines and TILs therapy [138].

#### 4.1.9. Starna Therapeutics, Immorna Biotherapeutics, and Everest Medicines

Starna Therapeutics (Suzhou, China) lead asset, STR-P004, is a tLNP/mRNA in vivo CAR-T therapy built on the company’s proprietary platform developed through screening of large lipid libraries to identify formulations enabling tissue-specific delivery. STR-P004 has entered clinical testing in relapsed or refractory (R/R) B cell non-Hodgkin lymphoma (NHL) (NCT07003178). Although limited information is available regarding the platform, including preclinical data, early clinical observations reportedly include profound B cell depletion after a single dose in a small number of lupus patients and in one NHL patient [139]. While trial enrollment criteria reference CD19-positive disease, the company has not, to our knowledge, formally disclosed the specific CAR target used.

Immorna Biotherapeutics (Hangzhou, China) is developing a targeted lipid complex nanoparticle system for mRNA delivery, designated JCXH-213, to enable in vivo generation of multicellular CAR therapies—including CAR-M, CAR-T, and CAR-NK cells. The candidate is currently being evaluated in an exploratory trial for R/R B cell NHL (NCT06618313) [78,140]. Despite the company’s announcement of first-patient dosing, detailed preclinical data, platform specifications, and specific CAR target have not been disclosed [141].

Everest Medicines (Shanghai, China) announced significant progress in its preclinical in vivo CAR-T program, which leverages a proprietary targeted LNP formulation with patented ionizable and stealth lipid components for mRNA delivery. The company reported that their platform achieved high T cell transfection, robust CAR expression, and effective B cell clearance in both humanized mouse and non-human primate models [142]. Further details regarding the technology, preclinical data, or target indications have not yet been disclosed.

### 4.2. LNP/circRNA Platforms Enabling Transient CAR Expression

#### 4.2.1. Sail Biomedicines

Sail’s lead program is an anti-CD19 in vivo CAR-T cell therapy based on the company proprietary tLNP/Endless RNA^TM^ (eRNA^TM^) platform, designed to transiently reprogram both CD8^+^ and CD4^+^ T cells with durable payload expression. Currently in preclinical development, Sail’s in vivo CAR-T drug candidate is advancing toward the clinic for evaluation in autoimmune diseases. 

At the core of this technology is Sail’s proprietary eRNA molecule, a circular form of translatable RNA in which the single-stranded RNA ring lacks both the cap and poly-A tail, sequences normally used by cells to regulate mRNA translation and degradation. The lack of the free 5′ and 3′ ends in the eRNA molecule makes it resistant to exonuclease degradation, thereby enabling superior payload stability and expression to maximize therapeutic window. Proprietary liver-detargeted LNP formulations improve endosomal escape, while incorporation of a single targeting moiety, specific to a common antigen expressed on both CD4^+^ and CD8^+^ subsets, enable selective delivery to T cells. In addition, modular conjugation chemistry allows rapid exchange of targeting moieties without altering the core LNP, making the platform highly adaptable. Leveraging this platform, Sail demonstrated that an optimized eRNA CAR sequence—developed through AI/ML-enabled screening of 5′ UTR, IRES, ORF, and 3′ UTR variants—achieved increased CAR expression and persistence in transfected T cells when compared to an ORF- and purification-matched mRNA CAR sequence. Treatment of total PBMCs or CD4- or CD8-depleted PBMCs in vitro with Sail’s drug candidate resulted in dose-dependent B cell killing within 48 h, confirming generation of functional CD4^+^ and CD8^+^ CAR-T cells. Consistent with this, a single in vivo administration of Sail’s drug candidate in tumor-free PBMC-humanized mice led to profound peripheral B cell depletion within 72 h across a wide dose range (0.01–1 mg/kg). In CD34-humanized models, in vivo administration of Sail’s drug candidate yielded superior % of CAR^+^ T cell (91%) and CAR molecules per cell (~2–6 × 10^3^) compared to tLNP/mRNA control. In a separate experiment using the same model, fractionated tLNP/eRNA dosing over two to three separate injections in a short cycle achieved deep B cell depletion in blood, spleen, lymph nodes, and bone marrow, performing better than the tLNP/mRNA control. Notably, repopulating B cells after depletion with Sail’s drug candidate exhibited a predominantly immature phenotype, supporting the potential for immune reset in autoimmune disease (ASCGT 2025, poster 774; 10th CAR-TCR Summit; personal communication) [143]. Collectively, Sail’s tLNP/eRNA platform enables potent and selective transient reprogramming of T cells in vivo. Their non-integrative, anti-CD19 in vivo CAR drug candidate is being advanced toward first-in-human studies for various autoimmune conditions. Beyond in vivo CAR-T, Sail’s pipeline includes several additional programs for autoimmune disorders (undisclosed payloads), in addition to eRNA programs focused on malaria [144].

#### 4.2.2. Orna Therapeutics

This company is developing a non-targeted, immunotropic LNP platform delivering proprietary circular RNA (oRNA^®^) encoding either anti-CD19 or anti-BCMA CARs to enable transient in vivo generation of CAR-T, CAR-NK, and CAR-myeloid cells (panCAR^TM^). Orna’s oRNA, generated via self-circularization and incorporating engineered IRES elements (SITC 2022, abstract 1222) [145], exhibits enhanced stability and durable translation in immune cells. Compared with mRNA-encoded CARs, oRNA-encoded constructs demonstrated higher and more sustained CAR expression in T cells and superior anti-tumor activity in vivo [146].

Using liver-detargeted immunotropic LNPs, identified through high-throughput screening in NHPs, oRNA-encoding reporters achieved >60% transfection of splenic T cells and >80% transfection of peripheral T, NK, and monocyte populations without hepatocyte targeting or the use of targeting ligands. Similar transfection efficiencies (~75%) were observed in CD34-humanized mice, demonstrating cross-species translation [146,147]. In the murine model, an LNP/oRNA anti-CD20 CAR induced rapid and robust B cell depletion (>75%) across blood, spleen, and bone marrow within 24 h, with sustained suppression for up to seven days. In NHPs, a single dose achieved ~95% peripheral B cell depletion, with durable effects (~82%) after one week (ASH 2024, abstract 3427) [147]. Subsequent studies demonstrated that a single 1 mg/kg dose of LNP/oRNA delivering a human/NHP cross-reactive anti-CD19 CAR in CD34-humanized mice, induced rapid and sustained B cell depletion across multiple tissues, with complete depletion achieved at doses as low as 0.03 mg/kg after repeat dosing. In a pristane-induced lupus model, anti-CD19 LNP/oRNA treatment significantly reduced B cell populations and anti-dsDNA autoantibodies, with efficacy comparable to or exceeding FDA-approved rituximab. In NHPs, repeat dosing induced dose-dependent B cell depletion across lymphoid tissues, followed by repopulation with predominantly naïve B cells by day 56 (ASH 2025, abstract 104) [146,148].

More recently Orna introduced its in vivo anti-BCMA panCAR program, demonstrating that three weekly doses (0.1 mg/kg) of LNP/oRNA encoding a human/NHP cross-reactive anti-BCMA CAR eliminated BCMA-expression tumor in humanized mice, outperforming an LNP/mRNA-delivered anti-BCMA CAR. In NHP, a single dose of anti-BCMA panCAR induced rapid and profound plasma cell depletion, reaching ~95% at 24 h, increasing to ~98% at 48 h, and remaining sustained for at least 72 h (ASH 2025, abstract 4117) [149]. Collectively, these data highlight the potential of Orna’s circRNA-based immunotropic LNP platform to enable transient, multicellular in vivo CAR therapies. Beyond Orna’s in vivo CAR program, the company pipeline includes gene-editing approaches for hemoglobinopathies, and vaccine development [150].

#### 4.2.3. Orbital Therapeutics

Orbital’s lead asset, OTX-201, is an optimized circRNA encoding an anti-CD19 CAR delivered via tLNP to generate CAR T cells in vivo. Currently in preclinical development for B cell-mediated autoimmune diseases, OTX-201 achieved complete B cell depletion in blood, spleen, and lymph nodes in NHPs, as reported at the 5th Annual mRNA-Based Therapeutics Summit in Boston [151]. It remains unclear whether further details on the targeting moiety, circular RNA architecture, or LNPs formulation have been disclosed, as we could not access the full conference abstract. In October 2025, BMS (Princeton, NJ, USA) acquired Orbital [152].

#### 4.2.4. Strand Therapeutics

This biotech company is also advancing an in vivo CAR-T platform based on long-acting circRNA encapsulated in non-targeted LNPs, currently in early preclinical development for hematological malignancies and autoimmune disease.

Strand’s circRNA technology employs proprietary IRES sequences enabling up to 10-fold higher payload expression in T cells compared with conventional IRES elements. Leukopak-derived mononuclear cells exposed to LNP/circRNA encoding an undisclosed CAR resulted in up to 80% of T and NK cells transfection, with lower levels observed in monocytes and B cells. These transfected cells exhibited strong cytotoxicity against multiple cell lines in vitro, and upon adoptive transfer into tumor-bearing mice, achieved robust tumor clearance comparable to that of lentiviral CAR-T cells. Using in silico modeling and high-throughput screening, Strand designed miRNA-responsive elements that de-target expression in non-desired tissues, while maintaining robust expression in target immune cells. This approach enabled more than 90% payload knock-down in the liver while maintaining high expression in other organs (ASGCT 2025, poster 781) [153]. In addition to the in vivo CAR-T program, the company is testing in the clinic a self-replicating mRNA-encoding IL12 medicine administered intratumorally (ASGCT 2025, abstract 394) [154] and other preclinical assets [155].

#### 4.2.5. RiboX Therapeutics

RiboX is also developing a T cell-targeted LNPs, formulated with a novel ionizable lipid, for in vivo delivery of an anti-CD19 CAR encoded by circRNA for B cell malignancies and autoimmune diseases. In vitro, circRNA-mediated transfection of activated primary human T cells demonstrated CAR expression for up to 9 days, whereas tLNP/circRNA transfection of non-activated human T cells achieved robust cytotoxicity against autologous B cells and CD19^+^ Raji cell line. Using a PBMC-humanized mouse model, a single intravenous dose of the tLNP/circRNA resulted in efficient CAR-T cells engineering, leading to significant B cell depletion in the spleen. Additionally, in PBMC-humanized mice bearing CD19^+^ Raji lymphoma xenografts, LNP treatment induced tumor remission (ASGCT 2025, abstract 1762) [156]. More recently, RiboX demonstrated that a single dose in NHP of their tLNP/circRNA encoding anti-CD19 CAR achieved remarkable B cell reduction in circulating whole blood, lymph node, bone marrow, and spleen (ACR 2025, abstract 0003) [157].

Beyond in vivo CAR-T, RiboX is developing therapies for ornithine transcarbamylase deficiency (ASGCT 2025, abstract 1508) [158], Duchenne muscular dystrophy (ASGCT 2025, abstract 1073) [159] and radiation-induced xerostomia and hyposalivation [160].

#### 4.2.6. Mote Therapeutics

Mote Therapeutics is developing a modular tLNP platform, referred to as Mobilize LNP, designed for extrahepatic delivery of circRNA-encoded CARs for hematologic cancers and autoimmune diseases. The platform allows single-step surface functionalization of LNPs with tissue-specific ligands via inline micromixing. In preclinical studies, T cell-targeted LNP/circRNA delivering GFP achieved ~55% transfection of T cells at low doses in humanized mice, while in NHPs, a single dose led to ~20% reporter expression in T cells without toxicity (ASGCT 2025, abstract 483) [161]. More recently, the company reported ~30% CAR^+^ CD8^+^ T cell generation and B cell depletion in NHPs, although further methodological details and the CAR target remain undisclosed [162].

### 4.3. LNP Platforms Enabling CAR Gene Integration, Supporting Durable Expression

#### 4.3.1. Stylus Medicine

Stylus Medicine is pioneering an elegant recombinase-mediated platform, delivered via tLNP, that enables efficient genomic insertion of large payloads into a safe-harbor locus. Currently, in preclinical development for B cell-driven malignancies, this strategy allows precise and durable in vivo generation of anti-CD19 CAR T cells following a single LNP administration.

At the core of this strategy are large serine recombinases (LSRs), enzymes that evolved in bacteriophages to insert viral genomes into host chromosomes without introducing double-strand breaks. Stylus has extensively engineered LSRs to optimize both the specificity and efficacy of transgene insertion into a safe harbor locus. Notably, payloads ranging from 3 kb to 13.5 kb were integrated with no loss of efficiency relative to size, highlighting the high-capacity nature of the platform (ASGCT 2025, poster 188) [163].

Building on this, human T cells were electroporated in vitro with DNA-encoded anti-CD19 CAR and mRNA-encoded LSR. Flow cytometry analysis demonstrated uniform and robust CAR expression in over 40% of T cells. Genome-wide integration analysis (GIA) and droplet digital PCR (ddPCR) confirmed precise and reproducible payload integration at the designated safe-harbor site with minimal secondary site activity. Functionally, the engineered CAR-T cell eradicated CD19^+^ Nalm6 B-ALL tumor and secreted IFNγ and IL2 upon antigen-stimulation in a dose-dependent manner. To evaluate the potential of LSRs for in vivo T cell reprogramming, anti-CD3-decorated LNPs were co-encapsulated with DNA-encoded anti-CD19 CAR and mRNA-encoded LSR. In PBMC-humanized mice that were previously implanted with Nalm6 tumors, a single intravenous LNP dose at 1 mg/kg induced CAR expression and integration in circulating T cells, accompanied by robust and durable anti-tumor effect out to 40 days. Treatment was well tolerated (ASGCT 2025, poster 797; personal communication) [164].

In summary, these findings demonstrate the feasibility of achieving efficient and durable in vivo CAR T cell generation with a single injection of a recombinase-based, LNP-delivered genome engineering strategy. Beyond its in vivo CAR-T approach, Stylus has not released information regarding its broader pipeline.

#### 4.3.2. Tessera Therapeutics

Tessera Therapeutics is pioneering a T cell–tLNP-delivered, all-RNA in vivo CAR-T cell therapy, currently in preclinical development for oncology and autoimmune disorders [165]. Based on their proprietary RNA Gene Writer technology, this platform enables stable, non-site-specific genomic integration of RNA payloads after a single LNP dose. [166]. Because both the Gene Writer enzyme and the payload are RNA-encoded, this approach eliminates the need for DNA templates, offering a versatile and potentially safer option for genetic medicine. At the core of this platform are Tessera’s RNA Gene Writers, engineered enzymes that harness the mechanism of target-primed reverse transcription (TPRT), in which a target genomic site is nicked to expose a priming site for reverse transcription of the RNA template, enabling insertions, deletions, or single-nucleotide substitutions without creating double-strand breaks [167,168]. Using proprietary LNP formulations, Tessera has demonstrated in vivo delivery of RNA Gene Writers and payloads across several tissues and disease models including correction of one of the mutations in the SERPINA1 gene (Serine Protease Inhibitor, group A, member 1) leading to alpha-1 antitrypsin deficiency restoring human PAH gene function in phenylketonuria, and repairing the HBB E6V mutation in sickle cell disease (ASGCT 2025, abstract 1632) [169]. To achieve tissue-specific payload delivery, novel LNP designs were developed. For example, a single intravenous dose of an HSC-targeting LNP resulted in 11-fold reduction in liver uptake compared to liver-tropic LNPs. Incorporation of a targeting moiety further enhanced specificity, increasing reporter expression in bone marrow by 55-fold (ASGCT 2025, abstract 668) [170].

These delivery and gene editing innovations provide the foundation for Tessera’s in vivo CAR-T cell therapy. In initial studies, in vitro transfection of human T cells with LNP-delivered Gene Writers and CAR templates generated functional anti-CD19 and anti-BCMA CAR T cells. Similarly, delivery of Gene Writers with T cell–tLNPs (undisclosed targeting moiety) enabled integration of an anti-CD20 CAR transgene into resting NHP T cells in vitro, achieving approximately 60% CAR^+^ T cells. Building on these findings, a single dose of T cell–tLNPs encoding an anti-CD19 CAR in tumor-free PBMC-humanized mice generated CAR-T cells that expanded overtime and reached an average of 24%, along with CD19^+^ tumors eradication compared with controls. In a CD34-humanized mouse model, delivery of an anti-CD20 CAR template yielded ~30% CAR-T cells and complete elimination of human B cells (ASGCT 2025, abstract 153 and personal communication) [171]. Together, these advances highlight the broad potential of Tessera’s RNA Gene Writer platform. Although proof-of-concept activity has been demonstrated with various CARs, Tessera’s lead in vivo CAR-T program remain undisclosed [165].

#### 4.3.3. NanoCell Therapeutics

NanoCell is developing a tLNP-based platform for in vivo CAR-T generation, using RNA-delivered Sleeping Beauty (SB100X) transposase to mediate non-site-specific genomic integration of DNA-encoded CARs. Its lead preclinical candidate, NCTX-01, is a dual anti-CD19/CD22 in vivo CAR-T therapy for B cell malignancies [172]. To enable efficient T cell engineering, NanoCell optimized an LNP formulation capable of co-delivering DNA and mRNA [36]. LNPs were then functionalized with both CD7- and CD3-specific binders. CD7 was selected for its broad expression on T cells and propensity for internalization [173], while anti-CD3 scFv was added to promote T cell activation and improved delivery. Dual CD7/CD3-targeted LNPs achieved superior transfection in resting T cells compared with single-targeted formulations and preferential uptake over NK cells and monocytes.

Using this platform, DNA encoding either anti-CD19 or dual anti-CD19/CD22 CARs was encapsulated into CD7/CD3-tLNPs (NCTX). In vitro, transfected T cells showed stable CAR expression for ~20 days and antigen-dependent cytotoxicity with IL-2 and IFNγ secretion. In PBMC-humanized mice bearing Nalm-6 tumors, a single NCTX dose resulted in dose-dependent tumor growth inhibition, with CAR expression reaching ~24% in spleen and bone marrow at higher doses of the dual CAR construct. (ASGCT 2025, abstract 213) [174]. In CD34-humanized mice, a single 0.05 mg/kg dose of NCTX-CD19/CD22 induced expanding CAR-T populations, robust tumor clearance (complete responses in 7/12 mice), sustained B cell depletion, and a marked survival benefit. Collectively, these data demonstrate that a single low-dose administration of NCTX can generate functional in vivo CAR-T cells capable of durable tumor control. NanoCell is advancing NCTX-01 toward clinical development for B cell malignancies, with exploratory efforts also underway in autoimmune indications [172].

#### 4.3.4. Cytiva

The company recently announced that it is leading a new initiative to develop an in vivo CAR-T cell therapy platform for gastrointestinal cancers. In collaboration with leading academic institutions, the program aims to create an LNP-based system for in vivo CRISPR genome editing to generate cancer-targeting CAR-T cells directly within the body [50,175].

### 4.4. Additional Non-Viral Platforms

#### 4.4.1. Velvet Therapeutics

Velvet is developing a non-targeted, cationic polymer-based synthetic nanoparticle platform for DNA delivery, referred to as DNP (DNA nanoparticle), which enables transient multicellular anti-CD19 CAR generation in vivo. Moreover, xNP/DNA encoding CARs against solid tumor antigens were tested in vitro, revealing Velvet’s intention to expand beyond B cell-mediated indications.

Velvet’s platform leverages the unique physical and biological properties of the cationic polymer/DNA polyplex. The poly(aspartic acid) derivatives polymer neutralizes the negative charge of DNA and compacts it into smaller nanoparticles with a reduced hydrodynamic radius and strong positive zeta potential, facilitating interaction with negatively charged cells and nuclear membranes. Uptake occurs primarily through clathrin-mediated endocytosis. To test biodistribution, C57BL/6 mice were injected with DNP encoding Luciferase-GFP reporter, and expression was observed in lungs, thymus, and lymph nodes. In a time-course experiment, expression was evident as early as 2 h—considerably fast for a DNA-based platform—and persisted for 60 h. Flow cytometry evaluation showed reporter expression in lymph nodes CD4^+^, CD8^+^ T cells and in B cells. To test B cell depletion, DNPs encoding a murine cross-reactive anti-CD19 CAR was injected twice into C57BL/6 mice. Progressive peripheral B cell depletion was observed throughout the experiment, with maximal reduction reached at study end (day 21), when the spleen also showed modest B cell reduction. Blood chemistry and mouse weight showed that the therapy was well tolerated. Finally, PBMCs transfected with DNPs encoding a novel CAR against a solid tumor antigen, showed antigen-dependent cytotoxicity upon co-culture with tumor cell lines (ASGCT 2025, poster 699; ASH 2025, abstract 4131; SITC 2025, abstract 198; personal communication) [176,177,178]. In summary, Velvet’s platform enables in vivo payload delivery to multiple immune cell types with lymphatic tropism. While initial proof-of-concept studies employed an anti-CD19 CAR, the company’s pipeline indicates a primary focus on solid tumors with currently three programs listed [179].

#### 4.4.2. Aanastra

Aanastra (Pacific Palisades, CA, USA) is advancing a T cell-targeted, peptide-based nanoparticle system for in vivo delivery of anti-CD19 CAR mRNA to T cells, currently in preclinical development for hematologic malignancies and autoimmune indications. Aanastra’s in vivo CAR-T program leverages its proprietary PEP-NP platform, which uses short amphipathic peptides to form stable nanoparticles capable of efficient and selective RNA delivery while minimizing liver uptake. Initial in vitro data showed that PEP-NP-transfected human CAR-T cells exhibit potent killing of CD19^+^ Nalm-6 upon co-culture. In vivo activity was assessed in C57BL/6 mice, where a single IV dose of CD3/CD5-targeted PEP-NP at 0.5–1 mg/kg, induced robust reporter expression in splenic and peripheral T cells, with 15–18% GFP^+^ T cells. In CD34-humanized mice, IV dosing on days 1 and 5 with PEP-NP delivering anti-CD19 CAR mRNA (1 mg/kg) resulted in rapid (>90% within 12 h) and sustained B cell depletion for up to 12 days, followed by B cell recovery by day 20. CAR expression reached 10–20% of T cells within 12 h and was maintained for at least 12 days. No liver, kidney, or metabolic abnormalities were observed, supporting safety and repeat-dosing potential (AACR 2025, abstract 6401 and ASGCT’s Advancing Cell + Gene Therapies for Cancer) [180,181]. Beyond in vivo CAR-T, Aanastra’s pipeline includes additional PEP-NP-based approaches, including a tumor suppressor rescue (e.g., p53) and oncogene targeting/editing (e.g., KRAS) therapies [182].

#### 4.4.3. Strm.bio

Strm.bio recently announced its entry into the in vivo CAR-T space by using their proprietary megakaryocyte-derived extracellular vesicle (MV) platform, although further details are not yet available [183]. Prior to this, the company presented preclinical data demonstrating that the MV delivery system can home to the bone marrow and deliver a DNA-encoded reporter to hematopoietic stem and progenitor cells in mice and NHP, showing good tolerability even after repeated dosing (ASH 2023, abstract 2253) [184].

## 5. Beyond In Vivo CAR-T

In the following sections we comment on platforms that aim to deliver CAR transgene to immune cells other than T cells for instance by using myeloid cell-tropic LNPs for macrophage and monocyte targeting. This strategy is pursued by companies such as Create Medicines (discussed above), Carisma Therapeutics/Moderna (Cambridge, MA, USA) and Liberate Bio. Moreover, we discuss a targeted polymer-based nanoparticle approach for in vivo CAR-NK cells generation.

Myeloid-tropic LNPs achieve preferential delivery primarily through lipid composition-driven biodistribution. Formulation parameters such as ionizable lipid pKa, PEG density, and helper lipid composition influence LNP interactions with serum proteins, leading to formation of a dynamic protein corona that modulates cellular interactions in vivo. This corona can engage receptors on monocytes, macrophages, and dendritic cells, including lipoprotein and scavenger receptors, promoting internalization through phagocytosis and macropinocytosis rather than receptor-mediated endocytosis alone. Collectively, these mechanisms bias mRNA delivery toward myeloid populations in vivo, enabling in vivo CAR-M engineering without the need for dedicated surface ligands [37,185].

### 5.1. Carisma Therapeutics

This Philadelphia-based company pioneered the field of CAR-myeloid cell therapies, providing the first demonstration that macrophage and monocyte effector functions can be redirected toward tumor antigens through CAR signaling. Initially developed as an ex vivo cell therapy, both preclinical [186,187] and early clinical studies [188] showed that CAR-macrophage exert potent anti-tumor activity, remodel the TME and promote activation of adaptive immune response in HER2-positive solid tumors. In 2022, Carisma and Moderna partnered to translate this approach into an in vivo modality using LNP/mRNA technology. Preclinical studies demonstrated that systemic administration of LNP/mRNA in CD34-humanized mice bearing metastatic HER2- or GPC3-positive tumors elicited robust anti-tumor activity (SITC 2023, abstract 1514; SICT 2024, abstract 329) [189,190], with myeloid cells emerging as the predominant CAR-expressing population following intravenous dosing. Despite the discontinuation of Carisma’ in vivo approach [191], the GPC3-targeted in vivo CAR-M program remains under development at Moderna [78].

### 5.2. Liberate Bio

Liberate Bio recently demonstrated that LNP/mRNA-generated in vivo anti-CD20 CAR-M can induce deep yet transient B cell depletion in nonhuman primates, providing the first clear evidence that B cells can be efficiently targeted through the monocyte–macrophage compartment rather than T cells (ASGCT’s Advancing Cell + Gene Therapies for Cancer) [192]. Liberate’s high-throughput, barcode-based RAPTOR platform enables direct in vivo screening of pooled LNPs in NHPs to identify vehicles capable of targeting extrahepatic immune cells. This approach yielded several LNPs with selective tropism for monocytes in blood and bone marrow, forming the basis for Liberate’s in vivo CAR-M strategy. Using two lead formulations to deliver anti-CD20 CAR mRNA in NHP, the company achieved ~99% B cell depletion after two well-tolerated doses (0.5 mg/kg) administered four days apart, with B cell counts returning to baseline by day 14 [192]. Building on these results, Liberate is advancing toward clinical development with programs in autoimmune disease, such as systemic lupus erythematosus and multiple sclerosis, and oncology, including relapsed/refractory (R/R) multiple myeloma [193].

### 5.3. ImmunoVec

This company recently announced the development of an in vivo anti-CD19 CAR-NK therapy using their targeted polymer-based DNA delivery nanoparticle with proprietary, cell type-specific promoters that exclusively deliver the therapeutic payload within the intended cells. This early-stage approach aims to deplete B cells that cause autoimmune disease [50,194]. Additionally, ImmunoVec is also advancing programs for rare immune deficiencies such as Wiskott–Aldrich syndrome and IPEX syndrome [195].

## 6. Emerging Strategies for Precision T Cell Targeting: EVs, Exosomes, and Antigen Recognition

In this section, we highlight recent academic advances in T cell-targeted delivery, including extracellular vesicle (EV)-based platforms and an alternative nanoparticle functionalization strategy that exploits MHC–peptide–TCR recognition to achieve antigen-specific T cell targeting.

EVs are naturally occurring carriers released by cells, which encapsulate proteins and nucleic acids as part of their biogenetic pathways [196,197]. From a drug delivery standpoint, EVs possess several advantageous properties, most notably, their inherently low immunogenic and reactogenic profiles, which arise from their endogenous lipid bilayers [197,198]. In addition, EVs can accommodate a wide range of cargos and allow payload loading through genetic manipulation of the producer cells [198,199]. Moreover, EVs cellular specificity can be tailored via engineering of the producer cells, enabling surface presentation of targeting moieties or fusogenic elements that direct tissue or cell-type tropism. Indeed, early proof-of-concept studies have shown that EVs displaying an anti-CD2 ligand can preferentially interact with lymphocytes, while incorporation of viral envelope glycoproteins can enhance membrane fusion and cellular internalization, supporting effective in vivo delivery of protein and nucleic acid cargos to T cells [200].

Nevertheless, several hurdles remain for EV-based delivery platforms, including intrinsic biological heterogeneity that hampers reproducibility, safety concerns, and the operational challenges of scaling cell-derived production systems to meet GMP standards for clinical translation. This lack of uniformity stems from the heterogeneous nature of EV preparations, which consist of vesicles that vary widely in size, origin, and molecular makeup [201].

Exosomes represent a well-characterized subclass of EVs, typically 30–150 nm in diameter and originating from the endosomal multivesicular body pathway, with properties that make them particularly attractive for gene delivery applications. These include high biocompatibility, relatively uniform nanoscale size, inherent cell tropism, viral-like transfection efficiency, stability in circulation, and low immunogenicity [202,203]. Exosomes can be engineered to improve cell- or tissue-specific targeting, either through ex vivo modification or through genetic programming of producer cells. A broad range of cargo-loading strategies has been explored, including passive incubation, genetic manipulation of producer cells to enhance endogenous cargo packaging, direct transfection of isolated exosomes, and physical methods such as electroporation or sonication to transiently permeabilize vesicle membranes [202]. Recent studies have demonstrated the feasibility of using engineered exosomes to deliver CAR-encoding mRNA to T cells in vivo. For example, Si et al. reported CD3/CD28-targeted exosomes activate T cells and facilitate intracellular delivery of CAR mRNA, resulting in functional CAR expression and anti-tumor activity in preclinical models [204].

An elegant strategy to direct LNPs to T cells, without relying on lineage markers, leverages antigen recognition to selectively engineer defined T cell populations in vivo. This approach exploits peptide–MHC recognition to achieve cell-type specificity at the level of T cell receptor [205]. In this context, antigen-presenting nanoparticles (APNs) represent a distinct strategy for antigen-specific T cell targeting in vivo. Here, lipid nanoparticles are functionalized with peptide–MHC (pMHC) complexes that engage cognate TCRs on disease-relevant T cells, promoting selective internalization and mRNA delivery through receptor-mediated endocytosis. In preclinical models, APNs have been used to selectively eliminate autoreactive T cells in type 1 diabetes by delivering pro-apoptotic mRNA to β-islet-specific CD8^+^ T cells, as well as to engineer virus-specific T cells with CAR-encoding mRNA for anticancer activity, achieving therapeutic effects comparable to ex vivo CAR-T cell therapies. Although still at an early-stage relative to more established delivery platforms, APNs exemplify how TCR-level specificity can expand the precision and scope of in vivo immune cell engineering [205].

## 7. Discussion: Benefits and Limitations of In Vivo CAR-T

In vivo CAR-T therapy is a strategy designed to address several key challenges associated with ex vivo CAR-T approaches; however, it also introduces its own distinct limitations. In addition, some challenges, as well as potential strategies to overcome them, are shared between both therapeutic methods (Table 3).

### 7.1. Absence of Lymphodepletion

In the ex vivo approach, lymphodepletion improves CAR-T expansion and persistence but is associated with increased infectious risk, prolonged cytopenias, and indirectly related with higher rates of CRS, owing to enhanced CAR-T proliferation and cytokine production [9,206]. In contrast, in vivo CAR-T approaches do not require lymphodepletion and may in fact be compromised by it, potentially offering a path to reduce adverse effects associated with lymphodepletion and excessive immune activation.

An additional advantage of avoiding lymphodepletion is the potential for epitope spreading. Following the initial CAR-T-mediated attack on tumor cells expressing the target antigen, damaged or dying tumor cells may release a broad array of additional tumor-associated antigens. In the context of an intact immune system, this antigen release may promote a more diverse and polyclonal anti-tumor immune response, potentially enhancing therapeutic durability and reducing the likelihood of antigen escape.

In vivo CAR-T strategies do not require in vitro cell activation and expansion. This feature substantially broaden the scope of CAR-based therapies beyond conventional T cells, enabling in vivo engineering of natural killer cells [27], myeloid cells [186,189,190,207], and other immune subsets [208], as discussed above. Furthermore, the absence of lymphodepleting conditioning opens opportunities to extend in vivo CAR technologies beyond oncology to non-malignant indications, such as autoimmune diseases, infectious diseases, and fibrotic disorders, where the toxicity and risks associated with lymphodepletion are unacceptable [209].

### 7.2. Immunogenicity

The presence of an intact immune system introduces the risk of immune responses against engineered cells, and the delivery platforms themselves, including LNPs and viral vectors [210,211]. Such immune responses may substantially reduce therapeutic efficiency.

LNP-based platforms generally exhibit lower immunogenicity than viral vectors [210,212]; however, their immunogenic profile varies depending on multiple factors, including particle size, surface charge, lipid composition, and route of administration, as reviewed [213,214]. Another important source of immunogenicity is the delivered mRNA itself, which may be recognized by intracellular pattern-recognition receptors, triggering innate immune activation and thereby reducing the efficiency of in vivo CAR-T generation [209].

### 7.3. Gradual Increase in CAR-T Cell Numbers In Vivo

In contrast to ex vivo CAR-T therapy, where a well-characterized and quantified cellular product is administered at a defined dose, in vivo CAR-T approaches result in a more gradual increase in the number of engineered cells over time [10,215]. Because higher CAR-T doses are associated with an increased risk of severe adverse effects [216], the absence of a sudden influx of large numbers of activated T cells may represent a safety advantage [206].

The transient nature of mRNA expression in non-viral delivery systems, and the ability to re-dose, offers an additional safety layer by modulating the dose regimen, therefore regulating number and persistence of CAR-T cells [78]. Unfortunately, the gradual increase in CAR-T number may limit therapeutic efficacy in rapidly progressing or highly aggressive tumors. Moreover, repeated dosing, whether using viral vectors or LNP-based platforms, may increase the risk of immunogenicity, potentially diminishing treatment efficiency over time.

### 7.4. Control of the Therapy

While the quality and quantity of ex vivo CAR-T products can be tightly controlled prior to administration, in vivo CAR-T therapies require fundamentally different, and more challenging, approaches to monitoring and regulation. Since the cellular product is generated directly within the patient, new strategies are needed to assess successful payload delivery, transfection efficiency, CAR expression, and the number, distribution, and activation status of CAR-T cells over time.

For LNP-based platforms, in vivo biodistribution is highly sensitive to chemical composition and structural modifications [37], and therefore typically requires the use of large animal models (e.g., NHP), as well as Sprague-Dawley rats. These species also provide a unique system for toxicity assessment, as they are particularly sensitive to LNP-associated toxicities [111,215]. Overall, the development of in vivo CAR-T therapies is likely to depend heavily on humanized models, advanced imaging and monitoring techniques, and long-term follow-up strategies to adequately assess efficiency and safety.

### 7.5. Efficiency and Safety

Non-integrating delivery platforms offer a major safety advantage over genome-integrating lentiviral vectors by eliminating concerns related to insertional mutagenesis and long-term consequences [215].

Although LNP-based platforms are generally associated with a lower risk of CRS and neurotoxicity compared with viral approaches, LNP-associated hepatic injury and inflammatory responses have been reported, especially with conventional LNPs formulations, and remain important safety considerations [215,217].

### 7.6. Common Challenges Shared by Ex Vivo and In Vivo CAR-T Therapies: Solid Tumors

Despite the strong potential of in vivo CAR-T strategies to overcome several limitations associated with ex vivo CAR-T manufacturing, the effective treatment of solid tumors remains challenging for both approaches. It is related to intrinsic characteristics of solid tumors, such as suppressive tumor environment, tumor stroma, and antigen heterogeneity.

In vivo CAR-T generation may reduce functional exhaustion by bypassing extensive ex vivo activation and expansion [10]. However, in vivo generated CAR-T cells are still exposed to the suppressive tumor microenvironment, which is known to induce and sustain T cell exhaustion [218,219,220]. In addition, patients with advanced cancers often exhibit reduced numbers of functional T cells and a compromised T cell repertoire due to prior therapies and chronic antigen exposure [11,221,222]. Thus, dysfunctional autologous T cells represent a shared limitation for both ex vivo and in vivo CAR-T approaches.

An additional, understudied challenge is the increased presence of highly suppressive intratumoral CD4^+^FOXP3^+^ regulatory T cells [11,223]. These cells may be unintentionally transfected during in vivo CAR-T delivery, further reinforcing local immunosuppression.

Conversely, the transient CAR expression achieved with non-integrating, mRNA-based platforms may limit chronic exhaustion [209]. In addition, the potential for antigen spreading, as discussed above, could promote the recruitment and activation of new, non-exhausted endogenous T cell clones, thereby partially compensating for CAR-T dysfunction.

The shortage of good antigen targets. Effective CAR-T therapy relies on tumor-specific surface antigens that, ideally, should be selectively, abundantly, and uniformly expressed on malignant cells and resistant to downregulation.

When target antigens are shared with healthy tissues, CAR-T therapy poses a significant risk of on-target, off-tumor toxicity, as reviewed [220]. In solid tumors, antigen expression is often low, heterogeneous, or unstable, facilitating immune evasion through transcriptional downregulation, epigenetic silencing, or antigen loss [11,220,224,225]. High intratumoral antigenic diversity further complicates target selection, and these limitations affect both ex vivo and in vivo CAR-T approaches.

Limited tumor homing and infiltration. Solid tumors are often protected by a dense stroma that forms physical and biochemical barriers to CAR-T cell infiltration, including extracellular matrix deposition, cancer-associated fibroblasts, and immunosuppressive immune cells [226]. In addition, unfavorable chemokine gradients further limit CAR-T trafficking to tumor sites [227,228,229].

Ex vivo CAR-T manufacturing typically enriches for CCR7^+^CD62L^+^ T cells, which favor homing to lymphoid organs and are beneficial in hematological malignancies but may be suboptimal for solid tumor targeting (as reviewed here [227]). In vivo CAR-T strategies could, in principle, preserve a more diverse T cell population with native homing and chemokine receptor profiles, potentially improving tumor trafficking. However, this potential advantage remains speculative and awaits experimental validation.

The challenges and limitations discussed above, whether unique to in vivo CAR-T approaches or shared with ex vivo CAR-T therapies, define key directions for future development in the field.

### 7.7. Modifications of Ex Vivo CAR-T: One Step Ahead

To improve efficacy and durability of CAR-T product, multiple modifications are actively being explored. Currently, these modifications are applied to ex vivo CAR-T approaches, but some of them have the potential to be further developed for in vivo CAR-T therapies. To overcome T cell exhaustion, new approaches include genetic disruption of exhaustion-associated genes [220] and modulation of exhaustion-related signaling pathways [230]. To increase safety, several strategies to regulate CAR-T cell activity are under development. These include, but are not limited to, advanced CAR designs such as pooled CARs and dual-CAR systems incorporating logical gating (“AND,” “OR,” and “IF–THEN” circuits), as reviewed elsewhere [10,11,220]. Split-CAR designs, which separate antigen-recognition and activation domains [231], may reduce the risk of relapse due to antigen escape. Moving forward, novel modular CAR design platforms have recently been reported, as reviewed [232].

Parallel advances aimed at improving safety include modifications of co-stimulatory domains [233], incorporation of suicide switches that trigger CAR-T apoptosis upon administration of inducing drugs [234], and elimination markers that enable depletion of CAR-T cells via antibody-dependent or complement-dependent cytotoxicity following administration of specific antibodies [235]. CAR-T activity may also be regulated by inducible CAR systems and reversible “on/off” control mechanisms [234,236,237]. Another highly promising direction is the development of “armored” CAR-T cells [238,239,240], capable of actively modifying the tumor microenvironment. These strategies include targeting the tumor stroma through expression of enzymes such as heparanase [241] and hyaluronidase [242], CARs directed against fibroblast activation protein [243,244], and CAR-T cells engineered to secrete immunostimulatory cytokines [10,245], all of which aim to enhance tumor accessibility and immune activation. Tumor homing and infiltration may also be improved by modulation of chemokine receptor expression [10], tumor tagging approaches, such as the use of probiotics [246] or FITC-conjugated amphiphile [247], and remodeling of tumor vasculature, as reviewed elsewhere [210].

Combination strategies represent another important avenue for improving efficacy. These include pairing CAR-based therapies with immune checkpoint inhibitors, immunomodulatory agents, or tyrosine kinase inhibitors, among others [11].

## 8. Future Directions

Looking ahead, next-generation in vivo CAR-T platforms are converging on several key areas of innovation, including advances in delivery technologies, identification of the most appropriate target antigens, improved selectivity, and optimization of RNA formats and chemistry. In addition, strategies enabling site-specific genomic integration of CAR transgenes into defined safe-harbor loci are emerging as a highly attractive but technically challenging direction aimed at improving safety.

Improving target selectivity remains a critical priority for future platform development. Off-target transduction of bystander cells, germ cells, inhibitory immune cells, and tumor cells may increase toxicity and raise significant safety concerns [10,225]. Future studies will also need to better define the most appropriate cell types—or even specific cellular subsets—to target [11], as discussed above for hematological vs. solid tumors.

Advances in delivery platforms will be central to overcoming challenges related to selectivity and efficiency. Ongoing efforts include further engineering of viral vectors to reduce immunogenicity while enhancing targeting specificity and transduction efficiency [17,71,248,249]. In parallel, the development of non-viral gene delivery systems is rapidly progressing, with continued optimization of LNPs [250], exploration of novel delivery materials, and incorporation of targeting antibodies or peptides to improve cell-specific delivery (reviewed here [214,251]).

For RNA-based platforms, persistence and efficiency of in vivo CAR-T cells may be further enhanced through optimization of the genetic payload, including the use of chemically modified nucleosides, self-amplifying RNA, and circular RNA constructs [214]. These approaches aim to extend CAR expression while maintaining favorable safety and immunogenicity profiles.

There is a growing trend in the field to develop strategies that enable safer integration of CAR transgenes into defined genomic safe-harbor loci, thereby minimizing the risk of insertional mutagenesis. To achieve site-specific insertion of large genetic payloads, companies are increasingly exploring alternatives to conventional CRISPR-based approaches, including retrotransposon- and recombinase-based genome engineering platforms [28,49,164]. However, these systems are inherently complex and often require large or multi-component genetic cassettes, posing significant challenges for packaging and delivery within a single vector.

Finally, emerging high-throughput and computational approaches are expected to play a transformative role in the field. Integration of multi-omics profiling, high-throughput screening platforms, machine learning models, and artificial intelligence-driven algorithms may enable systematic analysis of CAR delivery platform biodistribution and immunogenicity, improved characterization of the tumor microenvironment, and rational prediction of optimal vector and lipid nanoparticle design [206,252].

## 9. Conclusions

In vivo CAR-T therapy represents a novel and potentially transformative approach in cellular immunotherapy, offering solutions to several key limitations of conventional ex vivo CAR-T strategy. By enabling direct genetic programming of immune cells within the patient, this approach may simplify manufacturing, improve accessibility, and enhance safety, while expanding applicability beyond oncology. Although significant scientific, technical, and regulatory challenges remain, rapid advances in delivery platforms, CAR engineering, and computational tools are actively addressing these barriers. Together, these developments suggest the potential to reshape programmable immune therapeutics.

## Figures and Tables

**Table 1:** Viral delivery companies.

Company *	Delivery System	Genome Integration	CAR Target	Cell Target	Added co-Stimulation	Immunog. and Phago. Mitigation	Enrichment Strategy	Promoter	Stage
Umoja	LV	Yes	CD19, CD22, CD20 and NR	CD3	Yes	No	Yes	Yes	Clinical
Interius	LV	Yes	CD20, CD19 and NR	CD7	No	No	No	No	Clinical
EsoBiotech	LV	Yes	BCMA and NR	TCR	No	Yes (CD47^HI^; MHC-I^KO^)	No	Yes	Clinical data available
Kelonia	LV	Yes	BCMA	CD3	No	No	No	No	Clinical data available
Sana	LV	Yes	CD19	CD8	No	No	No	No	Preclinical
Genocury	LV	Yes	CD19	CD3	Yes	No	No	No	Clinical data available
Exuma	LV	Yes	CD19	CD3	No	No	No	No	Preclinical
Alaya.Bio	LV	Yes	CD19	CD3	No	No	No	No	Preclinical
Immunofoco	LV	Yes	NR	NR	NR	NR	NR	NR	Preclinical
Vyriad	LV	Yes	BCMA	CD3	No	No	No	Yes	Preclinical
Legend	LV	Yes	dual CD19/ CD20	NR	NR	NR	NR	NR	Clinical
Ensoma	VLP	Yes **	HER2 and NR	CD46	N/A	N/A	Yes	Yes	Preclinical
AAVivo	tAAV	No	CD19	NR	N/A	N/A	N/A	N/R	Preclinical
Azalea	EDV + AAV	Yes (site specific) ***	CD19, BCMA and NR	NR	N/A	N/A	N/A	No	Preclinical

The table summarizes companies developing viral vector-based delivery approaches, highlighting the delivery system, genome integration, CAR targets, cell targets, added co-stimulatory elements, immunogenicity and phagocytosis mitigation features, enrichment strategies, cell-specific promoters (promoter) and development stage (stage). * Companies’ locations are listed upon the first referral in the text. ** Genome integration enabled via co-delivery of Sleeping Beauty (SB100X) transposase. *** Genome integration enabled via CRISPR–Cas9 and homology-directed repair (HDR) template. Legend: NR, not reported; N/A, not applicable; VLP, virus-like particles; EDV, enveloped delivery vehicle; AAV, adeno-associated virus.

**Table 2:** Non-viral delivery companies.

Company	Delivery System	Genome Integration	Nucleic Acid Format	Molecular Strategy	CAR Target	Cell Target	Stage
Capstan	tLNP	No	mRNA	No	CD19	CD8	Clinical
MagicRNA	tLNP	No	mRNA	No	CD19	CD8	Clinical data available
Create *	tLNP	No	mRNA	No	TROP2; CD19	CD8	Preclinical
Aera	tLNP	No	mRNA	No	CD19	CD8	Preclinical
Nitto Denko	tLNP	No	mRNA	No	CD19	CD8	Preclinical
Kernal	tLNP	No	mRNA	No	NR	NR	Preclinical
Sanofi	tLNP	No	mRNA	No	CD19, CD22 and NR	CD8	Preclinical
Grit	tLNP	No	mRNA	No	CD19	NR	Preclinical
Starna	tLNP	No	mRNA	No	NR	NR	Clinical data available
Immorna	tLNP	No	mRNA	No	NR	NR	Clinical
Everest	tLNP	No	mRNA	No	NR	NR	Preclinical
Sail	tLNP	No	circRNA	No	CD19	CD4/CD8	Preclinical
Orna	LNP	No	circRNA	No	CD19; BCMA	Untargeted	Preclinical
Orbital	tLNP	No	circRNA	No	CD19	NR	Preclinical
Strand	LNP	No	circRNA	No	NR	Untargeted	Preclinical
RiboX	tLNP	No	circRNA	No	CD19	NR	Preclinical
Mote	tLNP	No	circRNA	No	CD19	NR	Preclinical
Stylus	tLNP	Yes (site specific)	DNA/mRNA	Yes (LSR)	CD19	CD3	Preclinical
Tessera **	tLNP	Yes (not site specific)	all-RNA	Yes (RNA GW)	NR	NR	Preclinical
NanoCell	tLNP	Yes (not site specific)	DNA/mRNA	Yes (SB100X)	dual CD19/CD22	CD7/CD3	Preclinical
Cytiva	(t)LNP	Yes	NR	CRISPR	NR	NR	Preclinical
Velvet ***	PolyN	No	DNA	No	NR	Untargeted	Preclinical
Aanastra	PN	No	mRNA	No	CD19	CD3/CD5	Preclinical
Strm.bio	MV	No	NR	No	NR	NR	Preclinical
Carisma ****	LNP	No	mRNA	No	GPC3	Untargeted	Preclinical
Liberate ****	LNP	No	mRNA	No	CD20	Untargeted	Preclinical
ImmunoVec ****	PolyN	No	DNA	No	CD19	NR	Preclinical

The table summarizes development stage, target cell types, CAR targets, molecular strategies, nucleic acid formats, site-specific versus random genome integration approaches, and the underlying delivery systems. * In addition to Create’s lead programs based on LNP/mRNA, the company is also developing CREATE which enables site-specific CAR gene integration into T cells. ** Tessera’s lead target undisclosed. Anti-CD19, anti-CD20, and anti-BCMA were used as POC only. *** Velvet’s lead targets against solid tumors undisclosed. Anti-CD19 CAR was used as POC only. **** Platform targets are either myeloid cells (Carisma and Liberate) or NK cells (ImmunoVec). Legend: NR, not reported; LSR, large serine recombinases; RNA GW, RNA Gene Writer; SB100X, Sleeping Beauty 100X transposase; circRNA, circular RNA; PN, peptide-based nanoparticle; MV, megakaryocyte-derived extracellular vesicle; and PolyN, polymer-based nanoparticle.

**Table 3:** Key advantages and limitations of ex vivo and in vivo chimeric antigen receptor (CAR) T cell therapy technologies.

	Advantages	Limitations
**Ex vivo CAR-T**		
1	Controlled and well-characterized cell product	Lymphodepletion required as a part of therapy
2	Proven by clinical data in thousands of patients	Severe adverse events (cytokine release syndrome, etc.)
3	Lack of systemic vector exposure	High requirements for clinical and manufacturing center capabilities
4	Predictable regulatory path with specific requirements	For some patients the therapy cannot be manufactured
5	Allogeneic products reduce cost and improve scalability	Immunogenicity risk, especially for the allogeneic products
**In vivo CAR-T: viral vectors**		
1	Gene delivery removes the need for cell manufacturing	Complex in-body biology: less control on phenotypes
2	Lower per-dose cost potential at scale	Off-target transduction and insertional mutagenesis risks (when using LVs)
3	Rapid therapy initiation, even for acute disease	Anti-vector immunity may limit repeat dosing
4	Long-term expression with integrating vectors	Difficult dose control and patient-to-patient variability
5	Established vector technology: LV, AAV, adenoviral platforms	
**In vivo CAR-T: LNPs**		
1	Fully non-viral and transient (when using mRNA or circRNA): better safety profile	Limited CAR expression may reduce therapeutic efficacy
2	Simpler manufacturing using mRNA processes	Limited in vivo cell specificity (when using untargeted LNPs)
3	Allows repeat dosing and finer control of CAR expression	Biodistribution/clearance are variable across species
4	Enable multiplex payloads: CAR, cytokines, switches	Lower technology maturity; fewer in-human data points
5	Potentially faster regulatory path and lower immunogenicity	Risk of toxic liver accumulation with conventional formulations

The table describes factors representing the research challenges related to each corresponding type of cell therapy.

## Data Availability

No new data were created or analyzed in this study. Data sharing is not applicable to this article.

## Abbreviations

The following abbreviations are used in this manuscript:

APNs	Antigen-Presenting Nanoparticles
ASGCT	American Society of Gene & Cell Therapy
CAR	Chimeric antigen receptor
CAR-M	CAR macrophages and monocytes; CAR myeloid cells
CAR-NK	CAR-Natural Killers
circRNA	Circular RNA
CREATE	CRISPR-enabled Autonomous Transposable Element platform
CRISPR	Clustered Regularly Interspaced Short Palindromic Repeats
CRS	Cytokine release syndrome
DARPins	Designed ankyrin repeat proteins
eRNA	Endless RNA
HSCs	Hematopoietic stem cells
ICANS	Immune effector cell-associated neurotoxicity syndrome
IEC-HS	Immune effector cell-associated hemophagocytic lymphohistiocytosis-like syndrome
iGPS	In Vivo Gene Placement System
IN	Intranodal
IP	Intraperitoneal
IRES	Internal ribosome entry sites
IV	Intravenous
L1	Human LINE-1 retrotransposon
LSRs	Large serine recombinases
LDL-R	Low-density lipoprotein receptor
LNP	Lipid nanoparticle
MCA	Multiple Correspondence Analysis
MDF	Multidomain fusion protein
MHC	Major Histocompatibility Complex
MM	Multiple myeloma
MND promoter	Myeloproliferative sarcoma virus enhancer, Negative control region deleted, dl587rev primer-binding site substituted synthetic promoter
MV	Megakaryocyte-derived extracellular vesicle
NHP	Non-human primates
NK	Natural Killers
oRNA	Circular RNA by Orna Therapeutics
PN	Peptide-based nanoparticle
POC	Proof of concept
PolyN	Polymer-based nanoparticle
R/R	Relapsed/Refractory
RACR	Rapamycin-activated cytokine receptor
scFvs	Single-chain variable fragments
SLE	systemic lupus erythematosus
TCR	T cell receptor
TU	Transducing Units
UTRs	Untranslated regions
VHH	Variable Heavy domain of Heavy chain
VLP	Virus-like particles
VSV	Vesicular stomatitis virus
VSV-G	VSV glycoprotein
xNP	Poly-asparamide-based synthetic nanoparticles by Velvet Therapeutics

## Appendix A

**Table A1:** Data set for Multiple Correspondence Analysis of 30 delivery platforms.

List of Companies	Delivery System	Genome Integration Capacity	Site Specific Editing	Nucleic Acid Format	Molecular Strategy	Cell Target	Inclusion of Co-Stimulation	Immunogenicity and Phagocytosis Mitigation Strategies	Selection/ Enrichment Mechanisms	Cell-Specific Promoters	Source of the Native Fusogenic Glycoprotein
Umoja	LV	Yes	No	ssRNA	No	CD3	Yes	No	Yes	Yes	Cocal
Interius	LV	Yes	No	ssRNA	No	CD7	No	No	No	No	VSV-G
EsoBiotech	LV	Yes	No	ssRNA	No	TCR	No	Yes	No	Yes	VSV-G
Kelonia	LV	Yes	No	ssRNA	No	CD3	No	No	No	No	VSV-G
Sana	LV	Yes	No	ssRNA	No	CD8	No	No	No	No	Paramyxovirus
Genocury	LV	Yes	No	ssRNA	No	CD3	Yes	No	No	No	NR
Exuma	LV	Yes	No	ssRNA	No	CD3	No	No	No	No	NR
Alaya.Bio	LV	Yes	No	ssRNA	No	CD3	No	No	No	No	VSV-G deficient; coated with PBAE
Vyriad	LV	Yes	No	ssRNA	No	CD3	No	No	No	Yes	VSV-G
Ensoma	VLP	Yes	No	DNA	Yes (SB)	CD46	No	No	Yes	Yes	N/A
AAVivo	tAAV	No	No	DNA	No	NR	N/A	N/A	N/A	N/A	N/A
Azalea	EDV + AAV	Yes	Yes	Protein/ sgRNA/DNA	Yes (CRISPR-Cas9)	NR	N/A	N/A	N/A	No	N/A
Capstan	tLNP	No	No	mRNA	No	CD8	N/A	N/A	N/A	N/A	N/A
MagicRNA	tLNP	No	No	mRNA	No	CD8	N/A	N/A	N/A	N/A	N/A
Create	tLNP	No	No	mRNA	No	CD8	N/A	N/A	N/A	N/A	N/A
Aera	tLNP	No	No	mRNA	No	CD8	N/A	N/A	N/A	N/A	N/A
Nitto Denko	tLNP	No	No	mRNA	No	CD8	N/A	N/A	N/A	N/A	N/A
Kernal	tLNP	No	No	mRNA	No	NR	N/A	N/A	N/A	N/A	N/A
Sanofi	tLNP	No	No	mRNA	No	CD8	N/A	N/A	N/A	N/A	N/A
Grit	tLNP	No	No	mRNA	No	NR	N/A	N/A	N/A	N/A	N/A
Starna	tLNP	No	No	mRNA	No	NR	N/A	N/A	N/A	N/A	N/A
Immorna	tLNP	No	No	mRNA	No	NR	N/A	N/A	N/A	N/A	N/A
Everest	tLNP	No	No	mRNA	No	NR	N/A	N/A	N/A	N/A	N/A
Sail	tLNP	No	No	circRNA	No	CD4/CD8	N/A	N/A	N/A	N/A	N/A
Orna	LNP	No	No	circRNA	No	Untargeted	N/A	N/A	N/A	N/A	N/A
Orbital	tLNP	No	No	circRNA	No	NR	N/A	N/A	N/A	N/A	N/A
Strand	LNP	No	No	circRNA	No	Untargeted	N/A	N/A	N/A	N/A	N/A
RiboX	tLNP	No	No	circRNA	No	NR	N/A	N/A	N/A	N/A	N/A
Mote	tLNP	No	No	circRNA	No	NR	N/A	N/A	N/A	N/A	N/A
Stylus	tLNP	Yes	Yes	DNA/mRNA	Yes (LSR)	CD3	N/A	N/A	N/A	N/A	N/A
Tessera	tLNP	Yes	No	all-RNA	Yes (RNA GW)	NR	N/A	N/A	N/A	N/A	N/A
NanoCell	tLNP	Yes	No	DNA/mRNA	Yes (SB)	CD7/CD3	N/A	N/A	N/A	N/A	N/A
Velvet	Polymer-based nanoparticle	No	No	DNA	No	Untargeted	N/A	N/A	N/A	N/A	N/A
Aanastra	Peptide-based nanoparticle	No	No	mRNA	No	CD3/CD5	N/A	N/A	N/A	N/A	N/A

Cytiva, Immunofoco, ImmunoVec, Legend, Strm.bio, Carisma and liberate platforms are not included in the analysis. Legend: NR, not reported; VLP, virus-like particles; EDV, enveloped delivery vehicle; AAV, adeno-associated virus; PBAE, poly β-amino ester biodegradable polymers; SB, Sleeping Beauty transposase, LSR, large serine recombinase; RNA GW, RNA Gene Writer, N/A, not applicable.
